# Development of an interpretable machine-learning model to predict insufficient functional improvement after aquatic therapy in stroke

**DOI:** 10.3389/fneur.2026.1871732

**Published:** 2026-10-06

**Authors:** Ying Chen, Jian Zhang, Shuyan Qie

**Affiliations:** 1 Department of Rehabilitation, Beijing Rehabilitation Hospital, Capital Medical University, Beijing, China; 2 Beijing Disabled Persons Rehabilitation Service Center, Beijing, China

**Keywords:** aquatic exercise therapy, Fugl-Meyer assessment, machine learning, Shapley additive explanations, stroke

## Abstract

**Background:**

Early identification of stroke survivors who are unlikely to achieve clinically meaningful motor improvement after aquatic therapy may help optimize rehabilitation prescriptions and support precision rehabilitation. This study aimed to develop and validate machine-learning models based on pretreatment Fugl-Meyer Assessment (FMA) data to predict insufficient motor improvement after aquatic therapy, to examine whether actual aquatic-therapy exposure improves prediction, and to identify the most important predictors.

**Methods:**

We conducted a single-center retrospective study of patients with stroke who received the routine aquatic rehabilitation programme in the Department of Rehabilitation Medicine between January 2022 and December 2025. The primary outcome was insufficient improvement in total Fugl-Meyer motor function at day 56 ± 7 days after the start of aquatic therapy, defined as Δ FMA <10 points; total FMA was recalculated as FMA upper-extremity plus lower-extremity scores. Temporal validation was used: cases from January 2022 to December 2024 formed the training set, and cases from January to December 2025 formed the temporal validation set. Two modeling strategies were developed. Model A included baseline clinical information and the initial rehabilitation and aquatic-therapy plans available after prescription and before the first aquatic session. Model B added actual treatment-execution parameters accumulated through day 14 (allowable landmark window, days 12–16). ExtraTrees, Random Forest, XGBoost, logistic regression and K-nearest neighbors were compared, and model performance was assessed with the area under the receiver operating characteristic curve (AUC), calibration, decision-curve analysis (DCA) and Shapley additive explanations (SHAP).

**Results:**

A total of 1,320 patients were included, of whom 409 had insufficient FMA improvement after aquatic therapy. The development set included 786 patients and 241 events, and the temporal validation set included 534 patients and 168 events. In the temporal validation set, Random Forest performed best for Model A, with an AUC of 0.818 [95% confidence interval (CI) 0.777–0.853] and Brier score of 0.172 (95% CI 0.160–0.186). At the frozen sensitivity-prioritized threshold of 0.408, Model A had an accuracy of 0.700, sensitivity of 0.857, specificity of 0.628, positive predictive value of 0.514, negative predictive value of 0.906 and F1 score of 0.643. Random Forest also performed best for Model B, with an AUC of 0.814 (95% CI 0.774–0.850) and Brier score of 0.174 (95% CI 0.163–0.186). The paired change in AUC was −0.004 (95% CI −0.022 to 0.014). Model A SHAP analysis highlighted planned aquatic-course duration, planned weekly aquatic frequency, baseline FMA-LE, baseline upper-extremity FMA and time from stroke onset to rehabilitation.

**Conclusions:**

The interpretable Model A used routine clinical data and initial rehabilitation prescriptions available before the first aquatic session and was evaluated in a later cohort from the same center. Model B analysis illustrates how risk could be updated at day 14 once genuine execution records become available. These analyses do not show that model-guided treatment adjustment improves motor recovery; external validation and prospective clinical-impact evaluation are required before clinical use.

## 1 Background

A central clinical question in post-stroke aquatic exercise therapy is not only whether the intervention is superior to conventional therapy on average, but also how to identify patients who may fail to achieve clinically meaningful functional improvement after treatment (1). If patients at high risk of insufficient improvement can be identified before treatment or early in the treatment course, rehabilitation teams may adjust aquatic prescriptions, combine them with land-based rehabilitation, and optimize treatment intensity, duration, and follow-up (2, 3). Conversely, recognizing limited benefit only after several weeks of therapy may delay treatment adjustment, increase patient burden and consume rehabilitation resources, thereby limiting the practical value of precision rehabilitation and individualized decision-making (1, 2).

Aquatic exercise has been used and recommended as part of post-stroke motor rehabilitation (4, 5). Buoyancy can reduce limb loading and fall risk, water resistance can provide adjustable load for active lower-limb exercise, walking and balance control, and a warm aquatic environment may help relieve pain, spasticity and fear of movement (5, 6). Randomized trials and systematic reviews suggest that aquatic exercise, either alone or combined with land-based training, may improve balance, gait, lower-limb function and physical performance after stroke (5–7). However, effects differ across outcomes and treatment protocols, indicating that aquatic therapy is not a uniform intervention with identical effects across all patients (4, 5).

Most previous studies of aquatic therapy after stroke have focused on group-average effects, comparing aquatic interventions with conventional rehabilitation on balance, gait or functional scores (5–7). Such studies are useful for judging whether aquatic therapy is beneficial at a population level, but they do not answer which patients remain at risk of insufficient improvement despite completing a routine course (1, 4). They also do not clarify whether initial dose information, completed sessions and cumulative exposure refine individual risk estimates, or how baseline motor function, disease course, neural pathway injury and concurrent rehabilitation exposure contribute to risk (8, 9). These questions are directly relevant to aquatic-therapy allocation, prescription intensity, expectation management, and precision rehabilitation pathways (1, 4).

Prediction modeling provides a different framework from conventional efficacy research (10, 11). Whereas intervention studies estimate average treatment effects, prediction models estimate an individual patient's risk of a clinically relevant outcome within a defined treatment context (12, 13). In aquatic stroke rehabilitation, insufficient motor improvement is a clinically meaningful risk state that aligns with prescription optimization (1, 9). Machine-learning methods can capture nonlinear associations among baseline function, rehabilitation dose, treatment execution, and other clinical variables (2, 9, 14). When combined with interpretable methods such as Shapley additive explanations (SHAP), they can also show the relative contribution and direction of predictors, improving the transparency of model outputs for rehabilitation teams (15, 16).

Taken together, the gap is not simply the absence of individual prediction after aquatic therapy. Previous aquatic studies have rarely combined an explicit prediction time, separation of initial prescription variables from fixed-landmark execution variables, later-calendar temporal validation, aquatic-dose and treatment-execution information, and interpretable threshold-specific reporting. Temporal validation, machine-learning prediction, and SHAP have each been used in broader stroke-rehabilitation research. Accordingly, the present study attempts to integrate these components within a temporally defined prediction framework for aquatic-rehabilitation outcomes.

Therefore, this study developed an interpretable Model A for insufficient Fugl-Meyer motor improvement using information available after the initial prescription and before the first aquatic session. Model B additionally incorporated treatment-execution information accumulated through days 12–16. We also identified aquatic prescription and treatment-execution variables associated with insufficient improvement, with the aim of informing precision aquatic rehabilitation and individualized prescription decisions.

## 2 Materials and methods

### 2.1 Study design and participants

This was a single-center retrospective study. Participants were all consecutive eligible patients with stroke who received aquatic rehabilitation in the Department of Rehabilitation Medicine, Beijing Rehabilitation Hospital, Capital Medical University, from January 1 2022 to 31 December 2025. No convenience sampling or selective inclusion was used. The study was approved by the institutional ethics committee. Because anonymized retrospective clinical data were used, the requirement for written informed consent was waived. Reporting followed TRIPOD and TRIPOD+AI guidance for clinical prediction models (12, 13).

Inclusion criteria were: (1) age 18–70 years; (2) ischaemic or haemorrhagic stroke confirmed by cranial CT or MRI; (3) first-ever stroke, disease duration of 1–6 months, and rehabilitation phase; (4) receipt of the routine aquatic rehabilitation programme; and (5) completion of FMA assessment within 1 week before aquatic therapy and at day 56 ± 7 days after treatment initiation. Exclusion criteria were: (1) recurrent stroke; (2) severe cognitive impairment, psychiatric disease or communication disorder that prevented FMA assessment or aquatic training; (3) missing baseline or day-56 outcome assessment; (4) severe musculoskeletal disease, peripheral neuropathy or other conditions that substantially affected motor assessment; and (5) another seriously incomplete record that prevented cohort definition.

If a patient had more than one eligible aquatic-rehabilitation course, only the first eligible course was retained. The 43 later-course records were removed before cohort analysis. Outcome values were not imputed. We excluded 21 records without a baseline FMA assessment, 40 without an FMA assessment in the day-56 ± 7-day window, and three with another incomplete clinical record; these reasons were mutually exclusive. Among the 1,320 included patients, HbA1c was missing in 59 (4.5%), LDL cholesterol in 67 (5.1%), MEP status in 104 (7.9%), MMSE score in 64 (4.8%), and HAMD score in 75 (5.7%). Supplementary Table S2 reports the number and percentage missing for every candidate predictor.

### 2.2 Aquatic rehabilitation records

All patients received the routine aquatic rehabilitation programme used in our department. Aquatic prescriptions were jointly formulated by rehabilitation physicians and uniformly trained aquatic therapists according to stroke type, disease duration, motor function, balance ability, cardiopulmonary tolerance, pain, spasticity, and safety risk (1, 4). Training contents included aquatic walking, underwater treadmill training, balance training, active lower-limb exercise, resistance exercise, and aquatic functional task training (5–7). Written departmental criteria specified intensity progression, intensity reduction and treatment stopping, while the content and progression rate remained individualized. Missed sessions, pauses, interruptions, protocol deviations and their reasons were recorded in the routine treatment record.

Aquatic-therapy variables were divided into two groups. The first group comprised initial prescription parameters available after the aquatic-therapy prescription was completed and before the first aquatic session, including time from stroke onset to aquatic-therapy initiation, planned session duration, planned weekly frequency, planned treatment duration in weeks, initial underwater treadmill speed, target Borg rating of perceived exertion, and prescribed water depth, temperature and flow/vortex intensity. These variables represented the initial plan rather than later revisions. Water temperature was recorded to the nearest 1°C, water depth in centimeters, and flow intensity as low, moderate or high.

The second group comprised treatment-execution parameters accumulated through day 14 after the first aquatic session, with an allowable landmark window of days 12–16. These parameters included sessions scheduled and completed by the landmark, cumulative aquatic-therapy time, landmark adherence, observed average session duration and weekly frequency, mean underwater treadmill speed, mean Borg score and maintained water depth, water temperature, and vortex intensity. Whole-course totals and information recorded after the landmark were excluded.

To capture the broader rehabilitation context, we also collected data on other routine rehabilitation treatments, including physical therapy (PT), occupational therapy (OT), speech and swallowing therapy (ST), gait training, balance training, robotic or body-weight-supported gait training, neuromuscular electrical stimulation, acupuncture, mirror therapy, conventional exercise therapy and home-exercise guidance (17, 18). For Model A, only nonredundant initial-plan variables, including planned weekly frequency and session duration, that were available before the first aquatic session were eligible (19–23); algebraically derived total-session and total-time fields were excluded. For Model B, corresponding execution summaries were truncated at day 14.

### 2.3 Outcome definition

The primary outcome was insufficient motor improvement after aquatic therapy. The Fugl-Meyer Assessment (FMA) includes upper-limb motor function (66 points) and lower-limb motor function (34 points), with a total motor score ranging from 0 to 100; higher scores indicate better motor function (24, 25). At baseline and follow-up, total FMA motor score was recalculated as FMA upper-extremity plus FMA lower-extremity scores. Change in FMA was defined as Δ FMA = follow-up FMA—baseline FMA. The 10-point cut-off was determined before model development using institutional clinical practice and published FMA measurement literature (26); it was treated as an operational responder threshold rather than a universal minimal clinically important difference. Therefore, Δ FMA < 10 points was defined as insufficient improvement, and Δ FMA >=10 points as clinically meaningful improvement. Because aquatic training mainly targets standing, walking, balance, and lower-limb control, a sensitivity analysis used change in the FMA lower-extremity score (FMA-LE; Δ FMA-LE = follow-up FMA-LE—baseline FMA-LE), with Δ FMA-LE < 6 points indicating insufficient lower-limb motor improvement (24, 27). All FMA assessments were performed by uniformly trained rehabilitation physicians or therapists. If multiple FMA scores were available within the same assessment window, the score closest to the prespecified time point was used.

Because the attainable change depends on baseline score, the frozen models were also evaluated in severe or very severe (baseline total FMA 0–55), moderate (56–79) and mild (80–100) strata. A ceiling-sensitive analysis excluded patients with baseline total FMA >90. These strata were used for exploratory performance reporting and not as claims of different treatment effects.

### 2.4 Candidate predictors

Candidate predictors were extracted from electronic medical records, rehabilitation treatment records, aquatic training records, neuroimaging reports, electrophysiological reports, and follow-up records. Variables were selected according to stroke rehabilitation guidelines, previous prognostic studies, clinical availability and the specific research question (1, 8, 9, 28, 29). Neuroimaging, electrophysiological and baseline motor variables were included because they are commonly associated with post-stroke motor recovery potential (30–34).

Candidate predictors included demographic and social factors [age, sex, body mass index (BMI), and years of education]; vascular risk factors and lifestyle factors (hypertension, diabetes, atrial fibrillation, smoking, and alcohol use); baseline stroke characteristics [stroke type, time from stroke onset to rehabilitation, time from stroke onset to aquatic therapy, baseline National Institutes of Health Stroke Scale (NIHSS) score, baseline FMA upper-extremity and lower-extremity scores and baseline modified Barthel Index (MBI)]; neuroimaging and electrophysiological indicators [lesion site, corticospinal tract involvement and motor evoked potential (MEP) results]; comorbid impairments and complications (shoulder pain or subluxation, dysphagia, dysarthria, spasticity, pain, cognitive impairment, depression, or anxiety); routine rehabilitation variables (initial planned frequency and session duration for PT, OT, ST, gait training, balance training, robotic or body-weight-supported gait training, electrical stimulation and acupuncture); and aquatic-therapy variables described in Section 2.2. Two trained researchers independently entered and cross-checked the data. Discrepancies were resolved by reviewing the original medical, aquatic-therapy or rehabilitation records.

Before feature selection, variables unavailable at the relevant index time and exact algebraic derivatives of retained variables were removed. Baseline total FMA was excluded because it equals FMA upper-extremity plus lower-extremity scores; final actual hospital length of stay was excluded because it was unavailable at the Model A index time. Remaining redundancy was screened using absolute Spearman correlation |ρ|≥0.90, and selection frequency was examined across 30 resamples. Supplementary Table S2 reports every source predictor, variable type, coding and reference category, missingness, measurement time, Model A or Model B eligibility, and its mapping to post-encoding model columns.

### 2.5 Sample-size estimation

Sample size estimation was conducted in accordance with Riley's criteria for clinical prediction model development and the classical 10-events-per-variable methodological principle (35). Based on institutional real-world data and relevant domestic studies, the baseline incidence of insufficient motor improvement after aquatic therapy was estimated at approximately 30%. The initial calculation indicated a minimum requirement of 536 patients and 161 outcome events, with a 15% allowance for case exclusion, missing data, and sample attrition. Unlike a fixed-predictor design, the present study adopted an exploratory feature screening strategy without predefining the number of final predictors. Therefore, model stability and generalizability were guaranteed through adequate overall sample and event volume, strict elimination of redundant variables, data-driven feature selection *via* cross-validation, and rigorous temporal validation using an independent later calendar cohort. The final analytical sample included 1,320 patients, among whom 409 patients presented insufficient FMA motor improvement after aquatic therapy. Both the total sample size and the event number substantially exceeded the prespecified methodological threshold, ensuring reliable model development and stable performance evaluation.

### 2.6 Data processing and model development

Temporal validation was used to split the dataset. Patients treated from January 2022 to December 2024 formed the training set (*n* = 786), which was used for model development; patients treated from January to December 2025 formed the validation set (*n* = 534), which was held out from preprocessing, feature selection, hyperparameter tuning and threshold selection and was used for final performance evaluation and SHAP visualization. Candidate predictors were preprocessed as follows: continuous variables were standardized when required by the classifier; categorical variables were one-hot encoded; numerical missing values were imputed with 5-neighbor K-nearest-neighbor imputation; and categorical missing values, including MEP, were imputed with the most frequent category. To avoid data leakage, imputation, standardization, encoding and recursive feature elimination (RFE) were fitted within each development-data training fold and then applied to its held-out fold (10–13).

Two machine-learning strategies were developed. Model A was a prescription-context model applied immediately after the initial rehabilitation and aquatic-therapy prescriptions were completed and before the first aquatic session. Its predictors comprised baseline clinical measurements and initial planned treatment variables available by that index time; revised plans, actual final hospital stay and delivered treatment were excluded. Model B was a landmark update applied at day 14 after the first aquatic session, with an allowable window of days 12–16. It added only execution information dated on or before the landmark and predicted the day-56 ± 7-day outcome, leaving approximately 6 weeks for reassessment.

The algorithms included ExtraTrees, Random Forest, XGBoost, logistic regression, and K-nearest neighbors. Logistic regression served as a conventional statistical comparator; ExtraTrees, Random Forest and XGBoost were used to capture potential nonlinear associations and interactions among candidate predictors (2, 9, 14). K-nearest neighbors provided a sample-similarity-based comparator. Because the outcome was moderately imbalanced, class weights were used for ExtraTrees, Random Forest, and logistic regression, the positive-to-negative class-weight ratio was adjusted for XGBoost, and the classification threshold was optimized for K-nearest neighbors (10, 11).

All models were trained in Python 3.9 using scikit-learn and XGBoost. RFE used a Random Forest ranking estimator with 120 trees, maximum depth 5, balanced class weights and feature_importances_; 20% of the remaining encoded feature columns were removed at each iteration. Candidate retained counts of 5, 10, 15, 20, 25, 30 and all eligible encoded columns were compared using stratified 5-fold development cross-validation. The smallest count whose mean AUC was within one standard error of the highest mean AUC was selected. After feature number was fixed, five-fold stratified cross-validation combined with grid search was used for algorithm-specific hyperparameter tuning. Table 1 reports both the search spaces and the selected configurations. Thresholds were derived from out-of-fold development probabilities: the Youden threshold was retained as a reference, and additional cut-offs targeting development sensitivity of at least 0.80, 0.85, and 0.90 were frozen before temporal validation. The final classifier for both Model A and Model B was Random Forest with n_estimators=500, max_depth=5, min_samples_split=5, class_weight=balanced.

**Table 1:** Hyperparameter search spaces and selected configurations.

Model	Hyperparameter search space
ExtraTrees	n_estimators={200, 500}; max_depth={5, 10}; min_samples_split={2, 5}
Random Forest	n_estimators={200, 500}; max_depth={5, 10}; min_samples_split={2, 5}
XGBoost	n_estimators={200, 500}; learning_rate={0.01, 0.05}; max_depth={3, 5}
Logistic Regression	C={0.1, 1.0}; penalty={l1, l2}
KNN	n_neighbors={9, 15}; weights={uniform, distance}

### 2.7 Model performance evaluation

Models A and B were evaluated in the validation set. Performance was reported across discrimination, calibration and clinical utility (12, 13). Discrimination metrics included AUC, accuracy, sensitivity, specificity, positive predictive value, negative predictive value, and F1 score, with 95% confidence intervals. For candidate-algorithm comparisons, each classifier's Youden reference threshold was derived from its own development-set out-of-fold probabilities and frozen before temporal validation; sensitivity-prioritized thresholds were reported only for the selected Random Forest classifier. Calibration metrics included the Brier score with 95% confidence interval, maximum calibration error, calibration slope, joint calibration intercept, calibration-in-the-large and calibration curves. Clinical utility was assessed with decision-curve analysis (DCA), which compares the net benefit of model-based strategies with treating all or treating none across threshold probabilities (12, 13). Algorithm selection was based on development-cohort cross-validated AUC; the temporal-validation cohort was reserved for evaluation. DCA and SHAP interpretation were performed for the selected algorithm in each modeling strategy.

### 2.8 Predictor-importance analysis

SHAP values were used to interpret the best-performing machine-learning models and to quantify the contribution and direction of key predictors (15). Larger absolute SHAP values indicate greater predictive importance (15). SHAP results were reported separately for Models A and B to examine predictor ranking, direction of effect, key parameter-risk relationships, and exploratory interactions between model parameters and baseline motor function, disease course or routine rehabilitation dose. Because correlated predictors can exchange rank or divide attribution, SHAP findings were interpreted at the level of predictor domains and not as independent or causal effects.

### 2.9 Sensitivity analysis

To assess robustness, we performed the following sensitivity analyses: (1) redefining insufficient improvement using the lower-extremity FMA outcome (Δ FMA-LE < 6 points); (2) evaluating the frozen models in severe or very severe, moderate and mild baseline-FMA strata; (3) repeating evaluation after excluding patients with baseline total FMA >90; (4) reporting validation performance at thresholds targeting development sensitivity of at least 0.80, 0.85, and 0.90; and (5) comparing model performance and SHAP rankings after adding day-14 execution variables.

### 2.10 Statistical analysis

SPSS 26.0 was used for data management and descriptive statistics. Continuous variables were summarized as mean ± standard deviation or median (interquartile range), and categorical variables as counts and percentages. Differences in baseline characteristics between the training and validation sets were used only to describe sample distribution and were not used for variable selection. Python 3.9 was used for model development, RFE, performance evaluation, DCA and SHAP analysis. Bootstrap resampling with 1,000 repetitions was used to calculate 95% confidence intervals for AUC, accuracy, sensitivity, specificity, positive predictive value, negative predictive value, F1 score, Brier score and calibration parameters. All statistical tests were two-sided, and *P* < 0.05 was considered statistically significant.

## 3 Results

### 3.1 Participant screening and baseline characteristics

A total of 1,458 records of patients receiving aquatic rehabilitation after stroke were screened. We removed 43 later-course records from patients with more than one eligible course, 21 records without baseline FMA, 40 without FMA in the day-56 ± 7-day window, three with another incomplete clinical record and 31 that did not meet the remaining eligibility criteria. Thus, 1,320 patients were included; 409 had insufficient motor improvement after aquatic therapy. The training set contained 786 patients, including 241 events, and the temporal validation set contained 534 patients, including 168 events. The participant-flow diagram is shown in Figure 1. The training and validation sets were broadly comparable in most demographic characteristics, comorbidities, baseline functional scores and major initial aquatic prescription parameters. Use of the later calendar cohort permitted evaluation under temporal changes in case mix and may better approximate later same-center deployment. Baseline characteristics are provided in Supplementary Table S1, and predictor-level missingness is provided in Supplementary Table S2.

**Figure 1:**
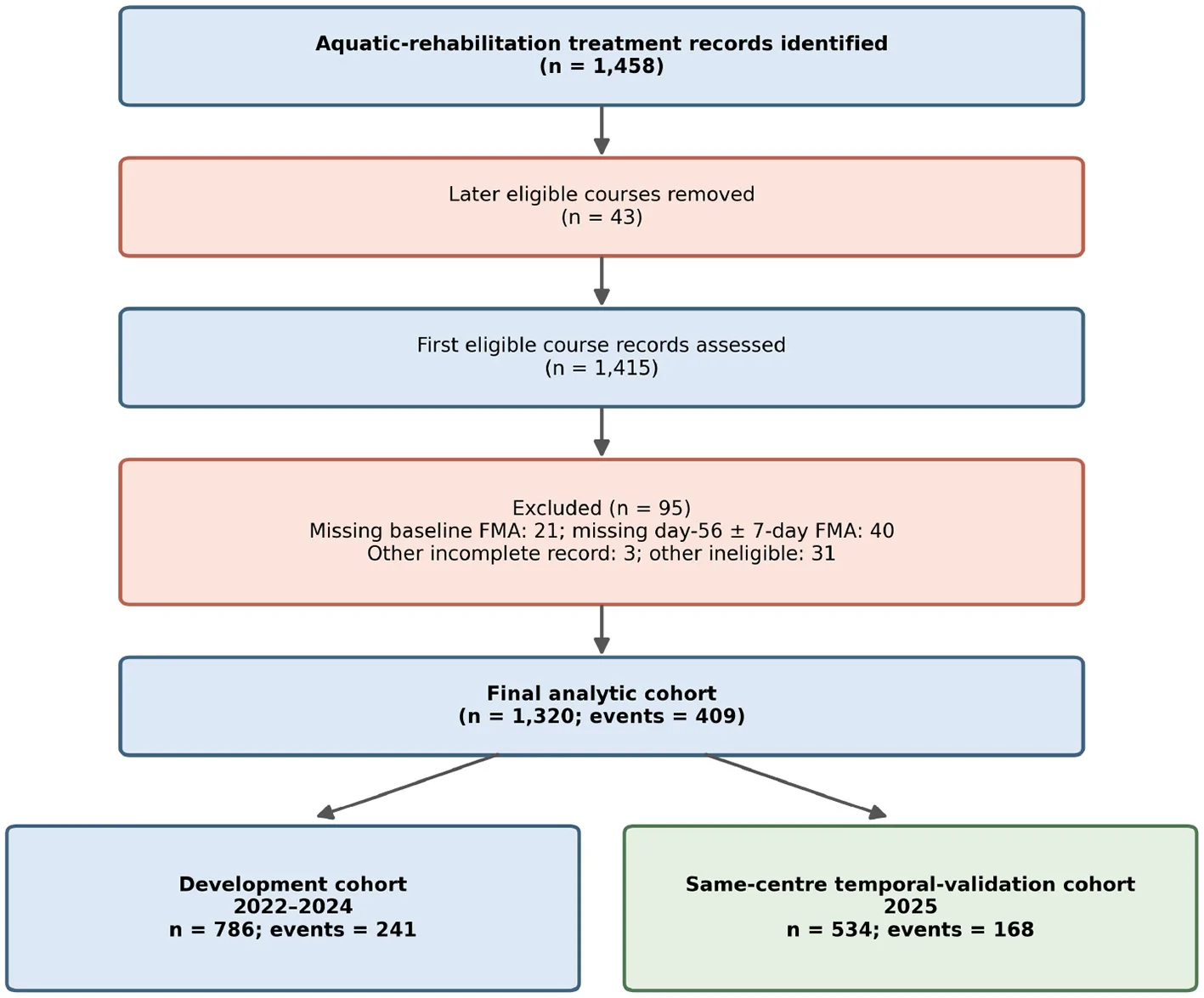
Participant screening flowchart.

### 3.2 Discrimination, calibration and clinical utility

Random Forest was selected for Model A by development cross-validation and achieved an AUC of 0.818 (95% CI 0.777–0.853) and Brier score of 0.172 (95% CI 0.160–0.186) in temporal validation. Across the five candidate algorithms, temporal-validation AUCs ranged from 0.769 to 0.818 and Brier scores from 0.172 to 0.195 for Model A, and from 0.756–0.814 to 0.174–0.189, respectively, for Model B; complete algorithm-specific discrimination, classification and calibration results are provided in Supplementary Tables S6B, C. At the Youden reference threshold of 0.435, Model A Random Forest accuracy was 0.713 (95% CI 0.674–0.751), sensitivity 0.839 (0.780–0.893), specificity 0.656 (0.609–0.700), positive predictive value 0.528 (0.490–0.567), negative predictive value 0.899 (0.864–0.930) and F1 score 0.648 (0.607–0.688). At the frozen sensitivity-prioritized threshold of 0.408, sensitivity increased to 0.857 (0.804–0.905) and specificity decreased to 0.628 (0.582–0.672). Random Forest was also selected for Model B by development cross-validation and achieved an AUC of 0.814 (95% CI 0.774–0.850); the paired change from Model A was −0.004 (95% CI −0.022 to 0.014). Table 2 reports all five candidate algorithms at their algorithm-specific frozen Youden thresholds and the selected Random Forest models at the sensitivity-prioritized operating point; receiver operating characteristic (ROC) curves and AUC forest plots are shown in Figures 2, 3. Complete results for the development sensitivity targets of at least 0.80, 0.85 and 0.90 are provided in Supplementary Table S5.

**Table 2:** Temporal-validation performance of all candidate algorithms at their frozen Youden thresholds and of the selected Random Forest at sensitivity-prioritized thresholds.

Strategy	Algorithm	Cut-off	AUC (95% CI)	Accuracy (95% CI)	Sensitivity (95% CI)	Specificity (95% CI)	PPV (95% CI)	NPV (95% CI)	F1-score (95% CI)	Brier score (95% CI)
Model A—Youden	Random Forest	0.435	0.818 (0.777–0.853)	0.713 (0.674–0.751)	0.839 (0.780–0.893)	0.656 (0.609–0.700)	0.528 (0.490–0.567)	0.899 (0.864–0.930)	0.648 (0.607–0.688)	0.172 (0.160–0.186)
Model A—Youden	Extra Trees	0.481	0.804 (0.761–0.842)	0.706 (0.667–0.743)	0.792 (0.726–0.845)	0.667 (0.620–0.708)	0.522 (0.480–0.561)	0.875 (0.839–0.907)	0.629 (0.582–0.670)	0.186 (0.175–0.199)
Model A—Youden	XGBoost	0.457	0.811 (0.772–0.847)	0.730 (0.693–0.766)	0.815 (0.756–0.863)	0.691 (0.645–0.735)	0.548 (0.508–0.590)	0.891 (0.858–0.919)	0.656 (0.612–0.696)	0.174 (0.159–0.190)
Model A—Youden	Logistic Regression	0.569	0.775 (0.731–0.814)	0.706 (0.668–0.742)	0.613 (0.542–0.685)	0.749 (0.705–0.790)	0.528 (0.476–0.580)	0.808 (0.778–0.837)	0.567 (0.511–0.617)	0.195 (0.180–0.213)
Model A—Youden	KNN	0.298	0.769 (0.726–0.812)	0.706 (0.667–0.742)	0.714 (0.643–0.780)	0.702 (0.656–0.743)	0.524 (0.479–0.567)	0.843 (0.810–0.875)	0.605 (0.554–0.650)	0.177 (0.161–0.194)
Model A—sensitivity-prioritized	Random Forest	0.408	0.818 (0.777–0.853)	0.700 (0.661–0.736)	0.857 (0.804–0.905)	0.628 (0.582–0.672)	0.514 (0.479–0.551)	0.906 (0.870–0.938)	0.643 (0.603–0.680)	0.172 (0.160–0.186)
Model B—Youden	Random Forest	0.438	0.814 (0.774–0.850)	0.719 (0.682–0.757)	0.786 (0.720–0.845)	0.689 (0.642–0.735)	0.537 (0.496–0.581)	0.875 (0.841–0.905)	0.638 (0.591–0.680)	0.174 (0.163–0.186)
Model B—Youden	Extra Trees	0.479	0.795 (0.751–0.833)	0.723 (0.685–0.762)	0.667 (0.595–0.732)	0.749 (0.705–0.792)	0.549 (0.500–0.605)	0.830 (0.798–0.861)	0.602 (0.546–0.654)	0.175 (0.162–0.190)
Model B—Youden	XGBoost	0.463	0.806 (0.767–0.841)	0.723 (0.685–0.760)	0.756 (0.685–0.815)	0.708 (0.664–0.751)	0.543 (0.500–0.588)	0.863 (0.830–0.893)	0.632 (0.586–0.675)	0.174 (0.159–0.190)
Model B—Youden	Logistic Regression	0.446	0.781 (0.738–0.818)	0.685 (0.644–0.725)	0.774 (0.708–0.833)	0.645 (0.596–0.694)	0.500 (0.460–0.543)	0.861 (0.824–0.894)	0.607 (0.562–0.648)	0.189 (0.173–0.208)
Model B—Youden	KNN	0.331	0.756 (0.711–0.795)	0.684 (0.644–0.721)	0.577 (0.506–0.649)	0.732 (0.683–0.773)	0.497 (0.446–0.550)	0.791 (0.760–0.821)	0.534 (0.477–0.590)	0.176 (0.163–0.190)
Model B—sensitivity-prioritized	Random Forest	0.441	0.814 (0.774–0.850)	0.719 (0.682–0.755)	0.780 (0.714–0.839)	0.691 (0.645–0.735)	0.537 (0.496–0.581)	0.872 (0.838–0.902)	0.636 (0.590–0.680)	0.174 (0.163–0.186)

**Figure 2:**
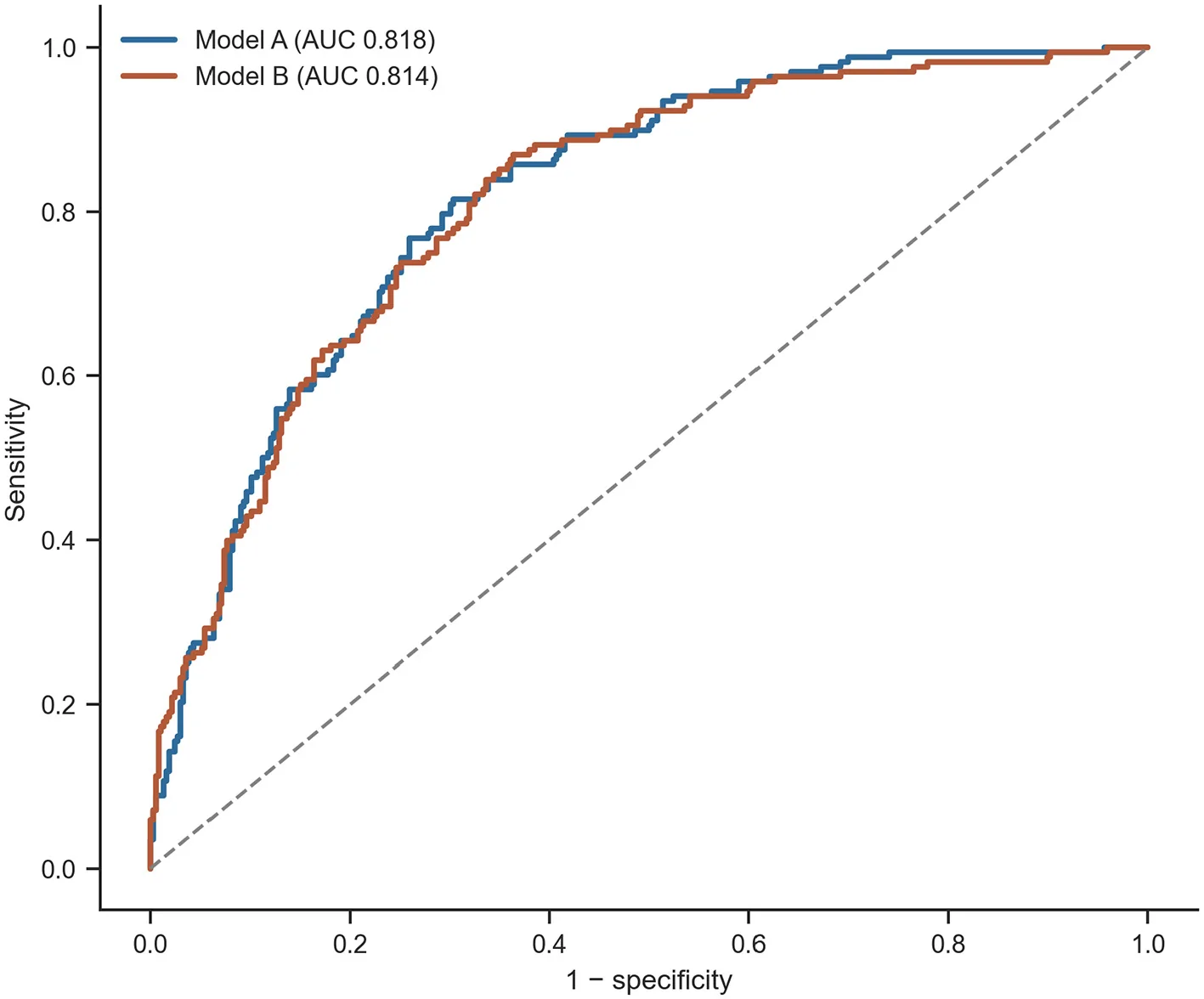
ROC curves of the selected models in the temporal-validation cohort.

**Figure 3:**
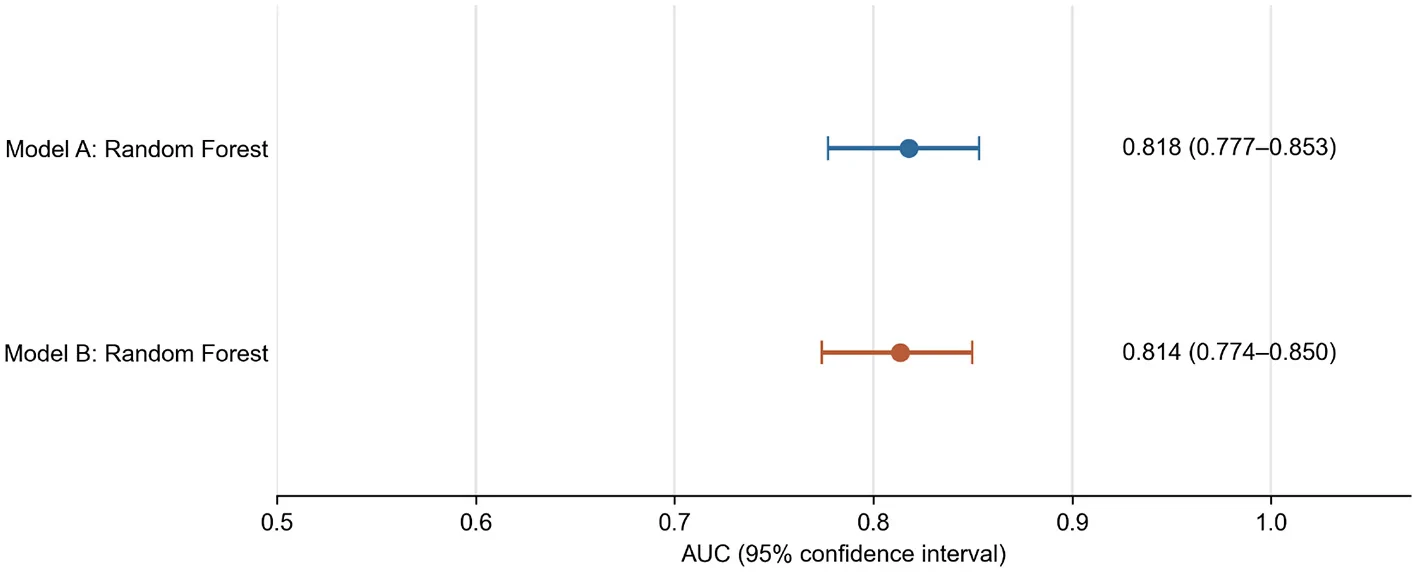
AUC forest plot of the selected models in the temporal-validation cohort.

Decision-curve analysis for Model A is shown in Figure 4. Model A had a calibration slope of 1.380 (95% CI 1.118–1.663), joint intercept of −0.594 (−0.708 to −0.485), calibration-in-the-large of −0.595 (−0.674 to −0.515) and maximum calibration error of 0.226. Model B had a Brier score of 0.174 (95% CI 0.163–0.186), calibration slope of 1.637 (1.343–1.976), joint intercept of −0.490 (−0.596 to −0.377), calibration-in-the-large of −0.544 (−0.607 to −0.480) and maximum calibration error of 0.197. Calibration curves are shown in Figure 5.

**Figure 4:**
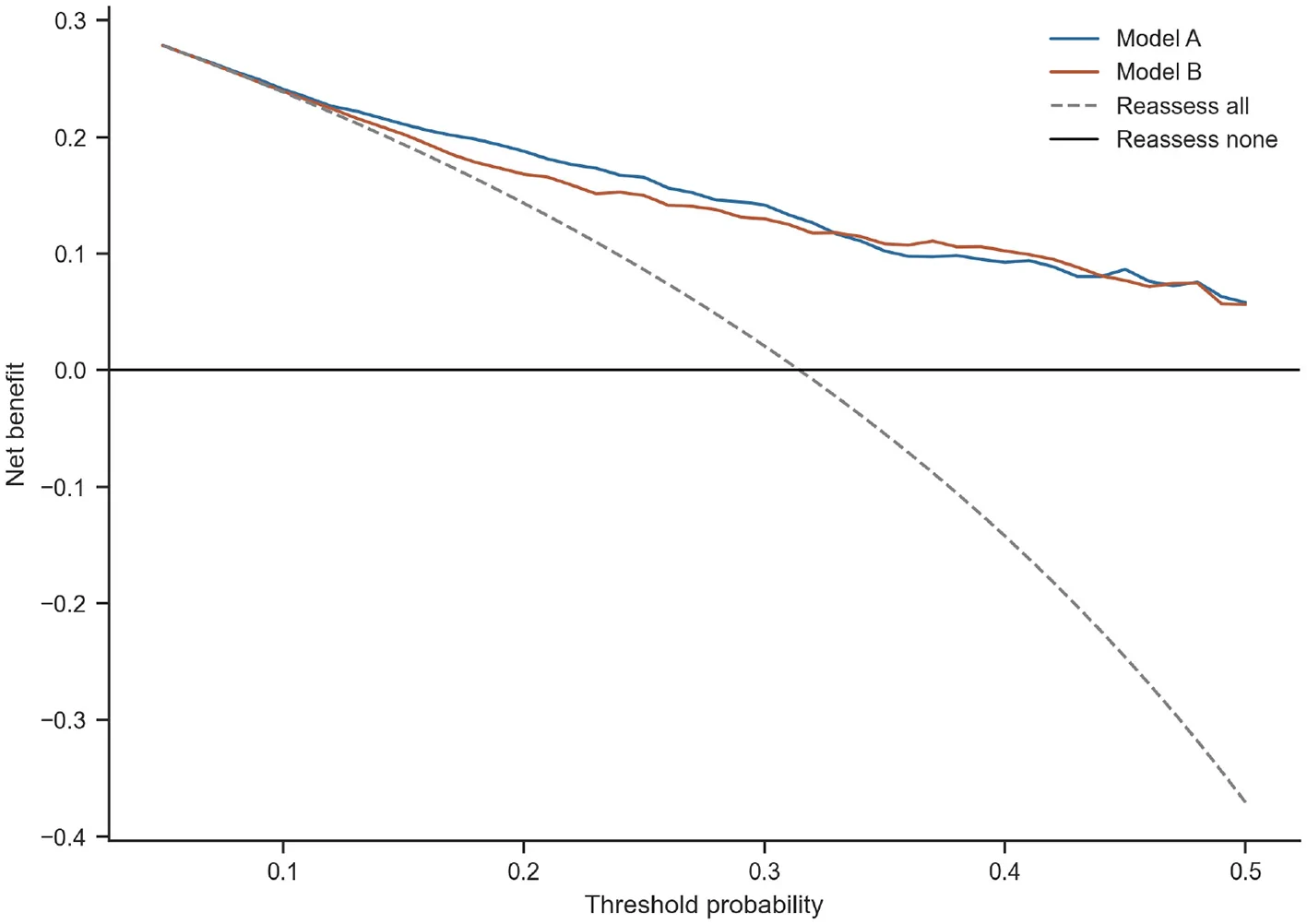
Decision-curve analysis of the selected models.

**Figure 5:**
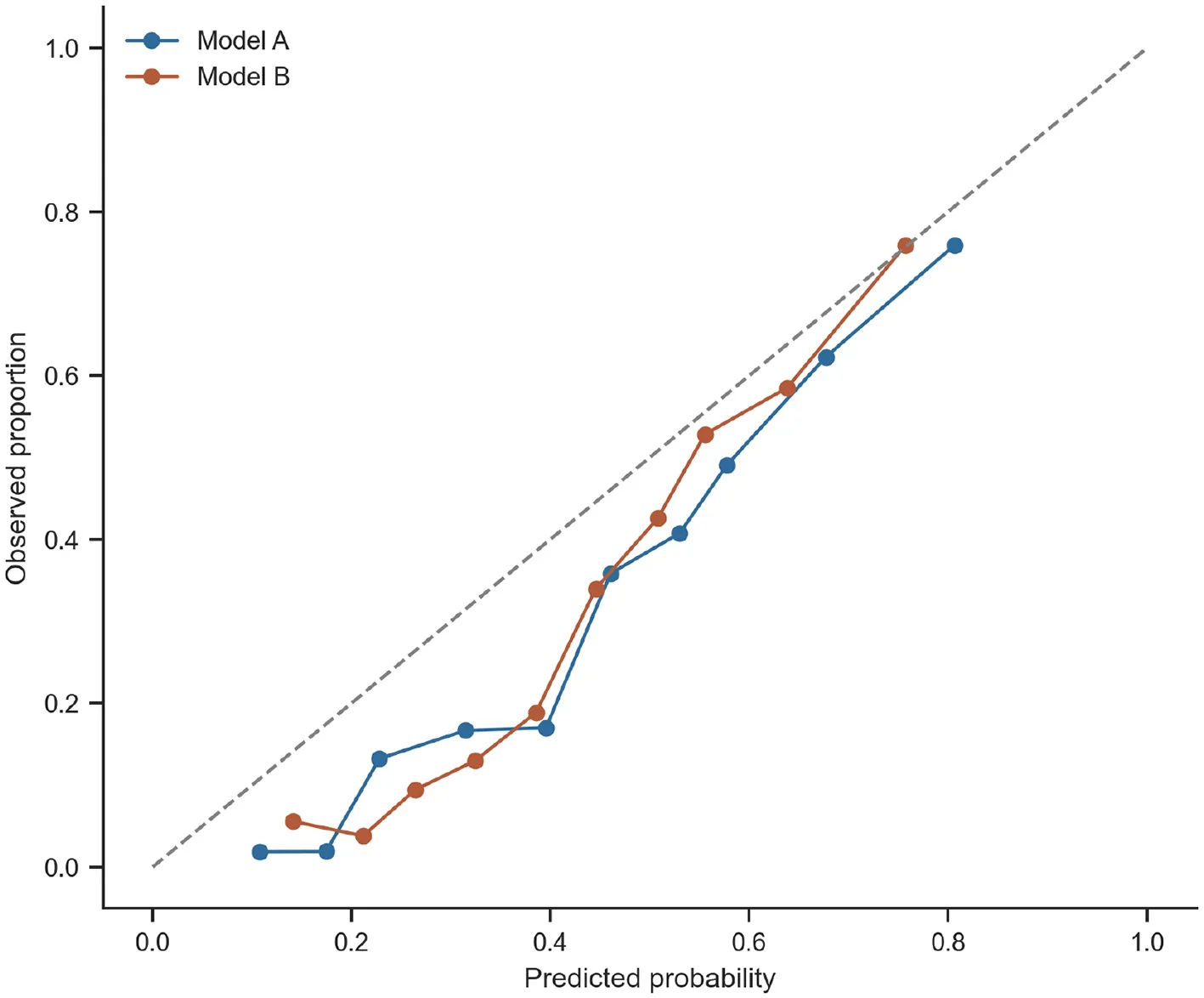
Calibration curves of the selected models in the temporal-validation cohort.

### 3.3 Feature selection and SHAP interpretation

RFE evaluated 5, 10, 15, 20, 25, 30, and all eligible post-encoding feature columns. The highest mean cross-validated AUC occurred with 15 features for Model A and 10 features for Model B; the one-standard-error rule selected 5 and 10 features, respectively. The best algorithm for both strategies was Random Forest. Model A retained time from stroke onset to rehabilitation, baseline FMA upper-extremity and lower-extremity scores, planned weekly aquatic-therapy frequency and planned treatment duration. Model B additionally retained aquatic sessions completed by day 14, mean aquatic treadmill speed, OT sessions completed by day 14, mean gait-training duration and mean neuromuscular electrical-stimulation duration. The selected encoded columns and their source-variable mappings are shown in Table 3.

**Table 3:** RFE-selected encoded features and source-variable mappings for Model A and Model B.

No.	Model A: Random Forest	Model B: Random Forest
1	Time from stroke onset to rehabilitation, days	Time from stroke onset to rehabilitation, days
2	Baseline FMA-UE	Baseline FMA-UE
3	Baseline FMA-LE	Baseline FMA-LE
4	Planned weekly aquatic-therapy frequency	Planned weekly aquatic-therapy frequency
5	Planned treatment duration, weeks	Planned treatment duration, weeks
6	—	Aquatic-therapy sessions completed by day 14
7	—	Mean underwater-treadmill speed by day 14
8	—	OT sessions completed by day 14
9	—	Mean gait-training session duration by day 14
10	—	Mean NMES session duration by day 14

SHAP analysis showed that planned aquatic-course duration, planned weekly aquatic-therapy frequency, baseline FMA-LE, baseline FMA upper-extremity score and time from stroke onset to rehabilitation contributed most to Model A output. In Model B, the highest contributions came from planned course duration, planned weekly frequency, aquatic sessions completed by day 14, baseline FMA upper-extremity score and baseline FMA-LE (Figure 6). These rankings illustrate how genuine day-14 data could later be interpreted; they do not provide evidence that changing treatment execution improves outcome. Development-cohort feature-count curves are shown in Figure 7. Because correlated predictors may exchange rank or divide attribution, SHAP values were interpreted as contributions to prediction, not as independent or causal effects (15, 16).

**Figure 6:**
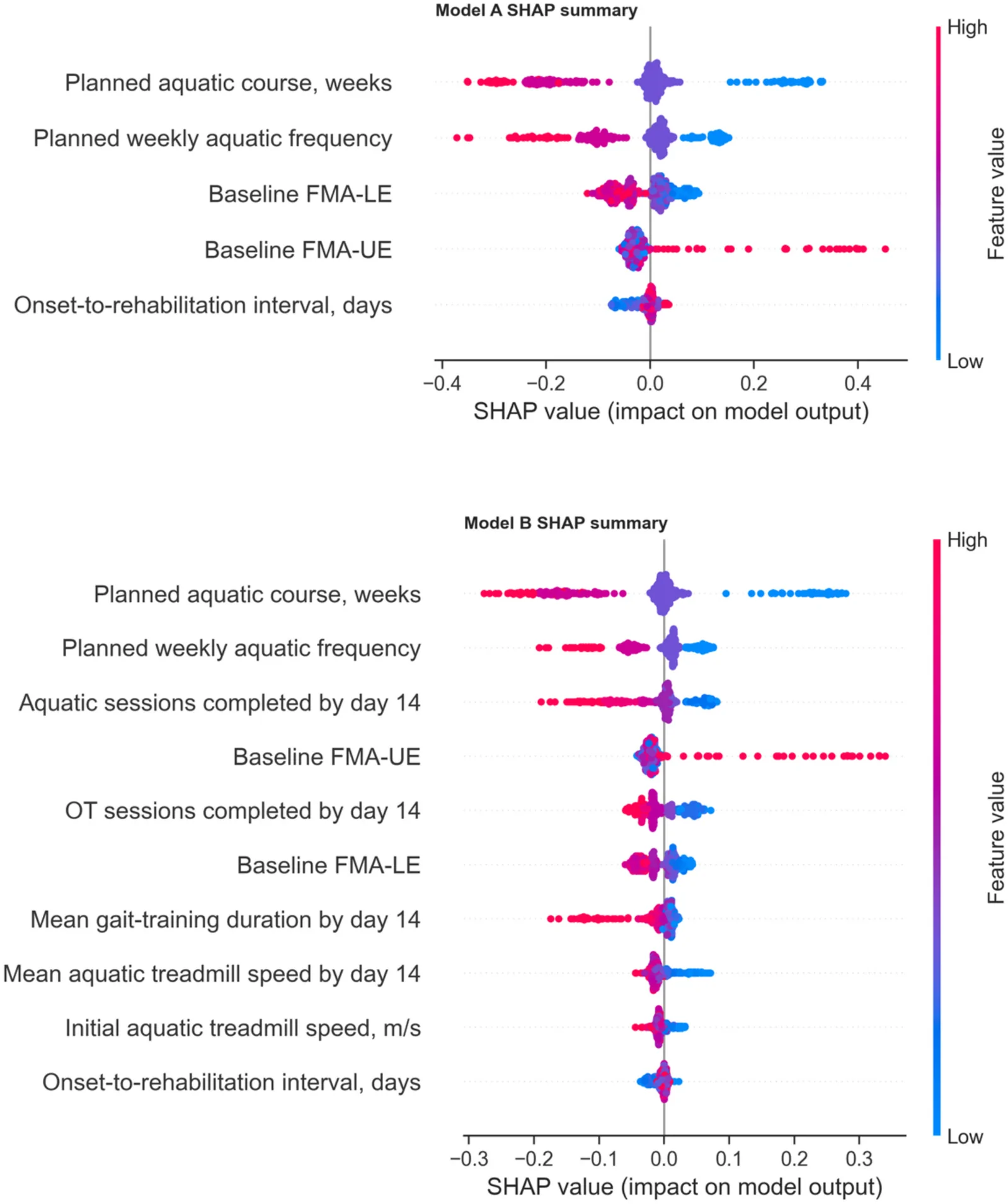
SHAP summary plots for Model A and Model B.

**Figure 7:**
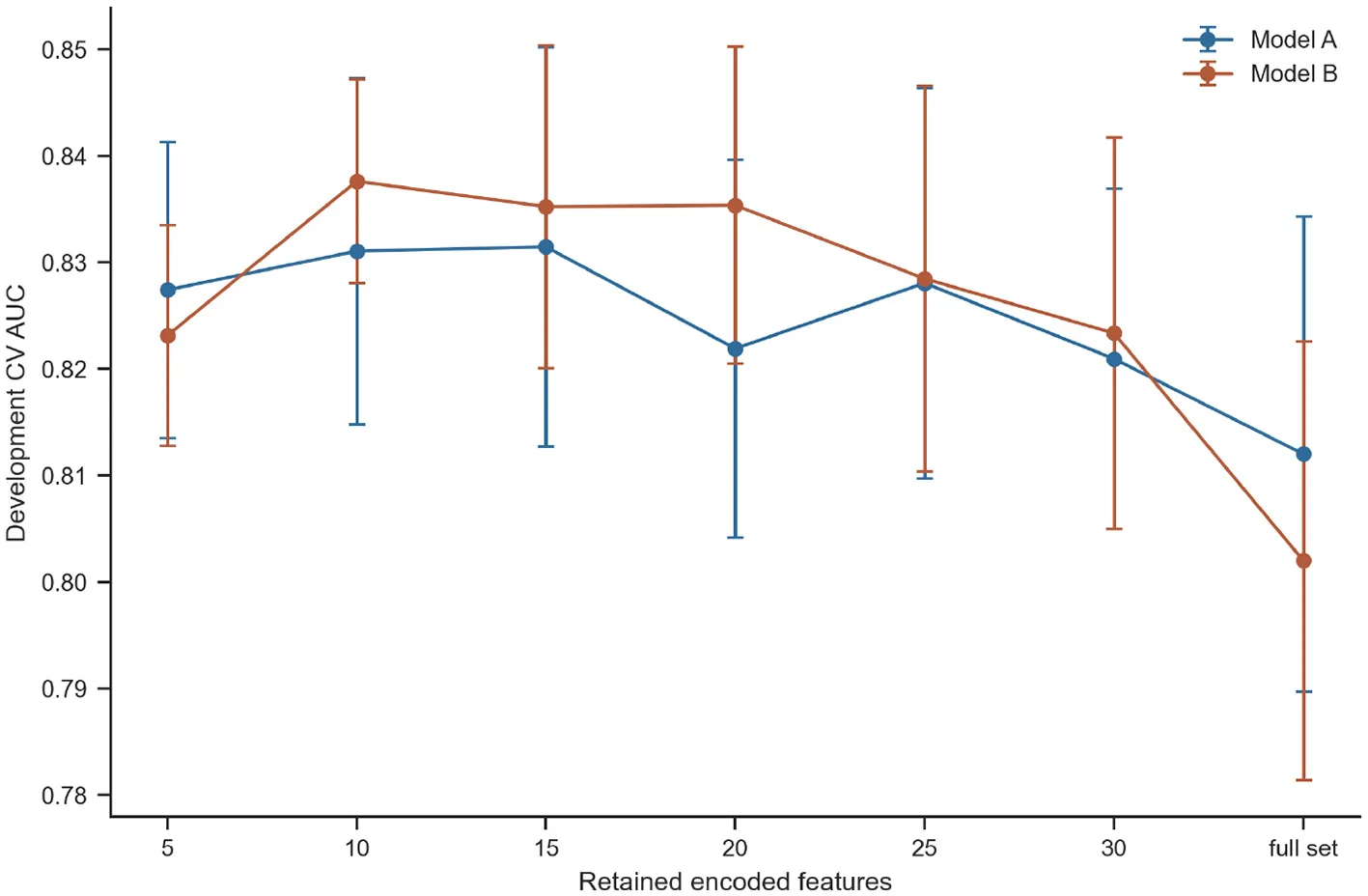
Development-cohort cross-validated AUC by retained encoded feature count.

### 3.4 Sensitivity analysis

When insufficient improvement was redefined as Δ FMA-LE < 6 points, Random Forest in Model A had an AUC of 0.781. Random Forest in Model B had an AUC of 0.779. Discrimination decreased under the alternative outcome, and Model B did not outperform Model A (Table 4). These secondary teaching analyses were treated as robustness checks rather than confirmatory tests.

**Table 4:** Sensitivity analysis using ΔFMA-LE <6 points as the alternative outcome.

Modeling strategy	Algorithm	AUC	Accuracy	Sensitivity	Specificity	Brier score
Model A	Random Forest	0.781	0.715	0.682	0.743	0.190
Model B	Random Forest	0.779	0.700	0.595	0.788	0.191

In the temporal-validation set, the severe or very severe, moderate and mild strata contained 222, 257, and 55 patients, including 80, 63, and 25 events, respectively. Model A AUCs were 0.800 (95% CI 0.741–0.860), 0.816 (0.757–0.870) and 0.825 (0.708–0.923); sensitivities at the frozen sensitivity-prioritized threshold were 0.875, 0.794, and 0.960. Seventeen patients had baseline total FMA >90, and all met the insufficient-improvement definition because a 10-point increase was mathematically unattainable. After excluding these patients, Model A had an AUC of 0.807 (95% CI 0.767–0.848), sensitivity of 0.841 and Brier score of 0.174. Corresponding Model B values are reported separately in the Supplementary materials.

## 4 Discussion

This study found that Model A, based on information available after the initial prescription and before the first aquatic session, achieved an AUC of 0.818 in the temporal validation set. At the frozen sensitivity-prioritized threshold, sensitivity was 0.857 and specificity was 0.628. The day-14 Model B pipeline achieved an AUC of 0.814 and a paired change in AUC of −0.004 (95% CI −0.022 to 0.014). Model A SHAP analysis indicated that baseline motor function, planned aquatic dose and time from stroke onset to rehabilitation jointly contributed to prediction. These findings support viewing aquatic therapy as one component of a comprehensive rehabilitation context rather than as an isolated fixed intervention with uniform effects across patients (1, 4, 5, 9, 17, 20).

Recent systematic reviews of aquatic exercise after stroke generally support its potential benefits for balance, lower-limb function and walking ability, although effects vary across outcomes (5–7). Randomized evidence and broader aquatic-exercise reviews suggest that aquatic training may improve postural stability and walking function (6, 36). Heterogeneity in disease stage, training content, and dose remains substantial (5, 7). The present findings help explain this heterogeneity: the outcome of aquatic therapy depends on baseline motor function, disease duration, neural pathway injury, exercise tolerance, actual completed dose, and concurrent land-based rehabilitation (1, 4). Thus, average-effect studies can show whether aquatic therapy may be useful at the population level, but they cannot replace individual risk identification and prescription adjustment (1, 9).

Machine learning is increasingly used to predict rehabilitation outcomes after stroke, but previous studies vary in sample source, outcome definition, predictor timing, validation design and risk of bias (9, 14). Some studies predict activities of daily living or overall functional status, whereas others use multimodal imaging, network injury markers or deep-learning methods to estimate recovery potential (8, 37–40). Compared with these studies, the present work used a clinically interpretable risk event–insufficient FMA improvement–rather than only predicting follow-up functional level. Temporal validation also made the validation setting closer to later same-center application (12, 13). The contribution is not temporal validation or SHAP alone, but the explicit separation of a pre-session prescription-time model from a fixed day-14 landmark workflow.

Model B did not improve discrimination over Model A, but this comparison was generated only to test the day-14 pipeline. In a future analysis, completed aquatic sessions and cumulative time could reflect adherence, tolerance, scheduling and actual exposure, whereas recoverability may already be partly captured by baseline motor function, disease duration, neurological injury and the initial prescription (1, 4). Initial prescription parameters are not neutral variables; they may embody clinicians' and therapists' judgments about safety, functional status, tolerance, and expected course. Therefore, Model B should not be interpreted as showing that more completed sessions are necessarily better. Rather, actual treatment execution appears to update risk estimates based on the initial assessment. Current stroke rehabilitation guidelines and dose studies emphasize that rehabilitation intensity should be determined by treatment timing, task content, specificity, tolerance and goals, not simply by minutes or number of sessions (1, 17, 20, 41).

Planned aquatic-course duration and weekly frequency had high SHAP importance in Model A. Completed aquatic sessions appeared among the leading features only in Model B and therefore cannot support a clinical dose-response claim. Research on gait training, task-specific training and rehabilitation intensity indicates that dose effects depend on task content, repetition quality, progression and patient engagement (19, 42–46). In patients with poorer baseline function, more severe corticospinal tract injury, pain, fatigue or poor cardiopulmonary tolerance, simply increasing aquatic-therapy sessions may not overcome biological or functional constraints (31, 33, 34). Conversely, patients with more severe impairment may be prescribed more intensive or longer therapy, producing confounding by indication in retrospective data (12, 13). Therefore, dose-related model patterns should be interpreted as predictive risk signals rather than causal dose thresholds.

Baseline FMA-UE and FMA-LE were retained in Model A and ranked highly in SHAP analysis, indicating that pretreatment motor status remains central to predicting clinically meaningful improvement after aquatic therapy (24–26). This is consistent with stroke-recovery research emphasizing baseline impairment severity, corticospinal tract integrity, brain-network injury and recovery potential (8, 28–30). However, baseline function can be interpreted in more than one direction. Patients with very low baseline function may have more theoretical room for improvement but may also be constrained by neural pathway injury and poor tolerance (31–33). Patients with higher baseline scores may function better but have difficulty reaching a fixed change-score threshold because of ceiling effects (26). Model A discrimination was similar across the three severity strata, although the mild estimate was imprecise. All 17 validation patients with baseline total FMA >90 met the insufficient-improvement definition because a 10-point increase was mathematically unattainable. Therefore, Δ FMA < 10 should be interpreted in relation to baseline FMA and should not be treated as a universal response criterion. When Δ FMA-LE < 6 was used in sensitivity analysis, AUC decreased to 0.781 for Model A, suggesting that lower-limb-specific insufficient improvement was more difficult to predict.

Neuroimaging and electrophysiological variables, including lesion location, corticospinal tract involvement and MEP results, were included because they are biologically linked to motor recovery potential (4, 30, 31). Recent studies continue to support the value of structural connectivity, functional connectivity, network injury, and multimodal biomarkers in predicting post-stroke motor recovery (8, 28, 29, 32). In this study, however, these variables were not among the most important SHAP predictors. This should not be taken to mean that neuroimaging or electrophysiology is unimportant. Possible explanations include relatively coarse routine clinical imaging and MEP records, missingness or information loss caused by categorical recording, and the fact that this study predicted 8-week insufficient improvement in an aquatic-therapy context rather than natural neurological recovery. The model is therefore best understood as a practical prediction tool based on routine clinical data rather than as a mechanistic model of neural repair (4, 9).

Several routine rehabilitation variables were considered because motor change during aquatic therapy occurs within a broader rehabilitation context rather than being driven solely by aquatic training (1, 17). OT sessions, gait-training duration, and neuromuscular electrical-stimulation duration entered the selected feature set only in Model B. These pipeline-test findings do not establish clinical associations. Recent evidence on gait training, balance training, dual-task training, robot-assisted gait training, exoskeleton training, neuromuscular electrical stimulation, task-oriented training, and occupational therapy suggests that these interventions may improve gait, balance, activities of daily living or motor function in selected stroke populations (18–22, 45–49). In routine practice, however, these variables may represent effective dose, baseline severity, compensatory allocation by therapists, adherence, or scheduling factors. Future studies should therefore consider them as predictive markers or contextual variables rather than direct causal levers.

A major barrier to clinical use of machine-learning models is limited interpretability (2, 14). SHAP analysis quantifies how each variable contributes to model output and helps rehabilitation teams understand whether a patient's elevated risk is driven mainly by poor baseline function, insufficient planned dose, incomplete treatment, or the structure of concurrent rehabilitation (15, 16). Nevertheless, SHAP results must not be interpreted as causal conclusions (15). A high SHAP value indicates that a variable helps discriminate risk within the model; it does not prove that changing the variable alone will reduce risk (15). This caveat is particularly important in rehabilitation research because treatment dose and patient status are closely intertwined. Thus, SHAP results are best used to generate clinical hypotheses–for example, whether patients with low baseline FMA-LE require different aquatic-dose progression strategies–rather than to define a uniform dose threshold.

Model A showed useful discrimination, but calibration indicated residual deviation. For screening, missing a patient with insufficient improvement may be more consequential than a false-positive referral; the sensitivity-prioritized threshold increased sensitivity from 0.839 to 0.857 while reducing specificity from 0.656 to 0.628. The preferred cut-off should therefore depend on the burden and safety of the downstream action rather than on the Youden index alone. However, the model should not be used as a stand-alone decision tool. A high-risk classification should first trigger verification of data completeness and adherence, repeat motor and functional assessment, and review of pain, fatigue, cardiopulmonary tolerance, and recovery trajectory. A multidisciplinary team may then consider individualized aquatic-dose or task-specificity adjustment, additional land-based gait or balance training, neuromuscular electrical stimulation or robotic training, and closer monitoring (1, 17, 41). These actions are proposed workflow options, not treatment effects demonstrated by this study. Model A applies before the first aquatic session; Model B only illustrates how reassessment could occur at day 14 after genuine execution data become available.

This study has several limitations. First, it was a single-center retrospective study, and patient source, treatment equipment, therapist experience, scheduling systems and local prescription habits may affect generalizability. Although temporal validation was used, the validation set came from the same institution; multicentre external validation is still needed (12, 13). Second, the routine aquatic programme was individualized and no formal protocol-fidelity audit was performed. Third, the fixed Δ FMA < 10 threshold has clinical interpretability but is affected by baseline severity and ceiling effects (26). Fourth, several laboratory, electrophysiological, cognitive and mood variables were missing; fold-specific imputation prevents leakage but cannot remove bias if missingness is informative. Fifth, routine clinical imaging and MEP records are less precise than research-grade quantitative imaging or standardized transcranial magnetic stimulation (TMS) assessment, which may underestimate the predictive value of neural pathway injury (4, 31). Sixth, correlated predictors may make feature selection and SHAP attribution unstable, and SHAP identifies model-association patterns rather than causal effects (15). Seventh, this study has not tested whether embedding the models in clinical workflow improves patient outcomes, so the models should not be claimed to improve aquatic-therapy efficacy directly.

Future work should proceed in several directions. First, multicentre external validation should be conducted across regions, hospital levels and aquatic-therapy equipment conditions to test transportability, and calibration stability (12, 13). Second, prospective studies should evaluate whether model-based risk stratification and prescription adjustment increase the proportion of patients who achieve clinically meaningful improvement. Third, future models should incorporate more granular dose variables, such as aquatic task type, repetitions, progression, Borg trajectories, pain and fatigue responses, and reasons for treatment interruption (5, 36). Fourth, quantitative neuroimaging, corticospinal tract lesion load, functional connectivity and standardized MEP indicators may improve biological interpretability (8, 30, 50–52). Finally, clinician-facing tools should present individual risk, key contributing variables, uncertainty, and suggested reassessment points so that models can serve precision aquatic rehabilitation and individualized prescription management (2, 13).

## 5 Conclusion

Using single-center data from patients receiving aquatic rehabilitation after stroke, this study developed and temporally validated an interpretable Model A. Baseline clinical information and initial rehabilitation and aquatic-prescription parameters available before the first aquatic session predicted insufficient motor improvement at day 56 ± 7 days. A day-14 Model B analysis illustrated how treatment-execution data could update risk once genuine records become available, but it did not provide clinical evidence about treatment execution. These findings are hypothesis-generating and do not show that model-guided treatment adjustment improves recovery. External validation and prospective clinical-impact evaluation are required before clinical deployment. Model outputs should support, rather than replace, the comprehensive judgment of rehabilitation physicians and therapists.

## Data Availability

The raw data supporting the conclusions of this article will be made available by the authors, without undue reservation.
