# Supplementary material for: Development of an interpretable machine-learning model to predict insufficient functional improvement after aquatic therapy in stroke
Source: Front Neurol. 2026 Oct 6;17:1871732. doi: 10.3389/fneur.2026.1871732 (PMC13681365; doi:10.3389/fneur.2026.1871732)
Supplement: Supplementary file 1 [file Supplementary_file_1.docx]

**Development of an Interpretable Machine-Learning Model to Predict Insufficient Functional Improvement After Aquatic Therapy in Stroke - Supplementary materials**

**Supplementary Methods S1. Routine aquatic rehabilitation programme**

Patients received the department's routine aquatic rehabilitation programme. Aquatic therapists had completed uniform departmental training, and written departmental guidance specified intensity progression, reduction, and treatment-stopping criteria. Rehabilitation physicians and therapists individualised treatment according to motor impairment, balance, cardiopulmonary tolerance, pain, spasticity, fatigue, and safety risk. Treatment could include aquatic walking, underwater treadmill training, balance tasks, active lower-limb exercise, resistance exercise, and functional tasks. Missed sessions, pauses, interruptions, and formal deviations were recorded.

**Observed initial-plan ranges**

| Parameter | Observed range |
| --- | --- |
| Planned aquatic session duration, min | 20.00–45.00 |
| Planned aquatic frequency, sessions/week | 2.00–5.00 |
| Planned aquatic course, weeks | 6.00–12.00 |
| Initial underwater-treadmill speed, m/s | 0.15–1.35 |
| Target Borg rating | 10.00–15.00 |
| Prescribed water depth, cm | 90.00–130.00 |
| Prescribed water temperature, °C | 32.00–36.00 |

**Supplementary Methods S2. Model-development and validation pipeline**

Total FMA motor score was corrected as FMA-UE plus FMA-LE. The positive outcome was a corrected total FMA increase below 10 points at day 56 ± 7 days. Model A was indexed before the first aquatic session. Model B was indexed at day 14 and used only execution data dated on or before day 14. Patients treated in 2022–2024 formed the development cohort; 2025 patients formed the same-centre temporal-validation cohort.

Numeric predictors were imputed with fold-specific KNN imputation (k=5) and standardised. Categorical predictors were imputed with the training-fold mode and one-hot encoded. Exact part–whole and algebraic redundancy was removed before modelling. Remaining numeric redundancy was screened using absolute Spearman correlation at 0.90; no additional variable exceeded that threshold after deterministic exclusions.

RFE used a Random Forest ranking estimator (120 trees, maximum depth 5, balanced class weights), feature_importances_, and a 20% elimination step. Feature counts of 5, 10, 15, 20, 25, 30, and all were evaluated by stratified 5-fold development cross-validation. The smallest count within one standard error of the maximum mean AUC was selected. After feature count was fixed, ExtraTrees, Random Forest, XGBoost, logistic regression, and KNN hyperparameters were compared by a separate 5-fold development cross-validation. Thresholds were selected from out-of-fold development probabilities and frozen before temporal validation.

Validation confidence intervals used 1,000 stratified patient-level bootstrap resamples without refitting the model or threshold. Calibration slope and joint intercept were estimated by logistic recalibration; CITL used the prediction logit as an offset. DCA evaluated threshold probabilities from 0.05 to 0.50. Software: Python 3.9, scikit-learn 0.24.1, XGBoost 2.1.4, SHAP 0.39.0, NumPy 1.24.4, pandas 1.2.4, and SciPy 1.10.0.

**Supplementary Table S1. Baseline and initial-plan characteristics**

| Variable | Development cohort (n=786) | Temporal-validation cohort (n=534) |
| --- | --- | --- |
| Age, years | 55.88 ± 8.22 | 55.78 ± 8.27 |
| Sex | Male: 476 (60.6%); Female: 310 (39.4%) | Male: 323 (60.5%); Female: 211 (39.5%) |
| BMI, kg/m² | 25.05 ± 3.02 | 25.05 ± 2.81 |
| Education, years | 10.58 ± 3.21 | 10.44 ± 3.23 |
| Hypertension | No: 535 (68.1%); Yes: 251 (31.9%) | No: 345 (64.6%); Yes: 189 (35.4%) |
| Diabetes mellitus | No: 643 (81.8%); Yes: 143 (18.2%) | No: 449 (84.1%); Yes: 85 (15.9%) |
| Atrial fibrillation | No: 745 (94.8%); Yes: 41 (5.2%) | No: 491 (91.9%); Yes: 43 (8.1%) |
| Stroke type | Ischaemic stroke: 592 (75.3%); Haemorrhagic stroke: 194 (24.7%) | Ischaemic stroke: 372 (69.7%); Haemorrhagic stroke: 162 (30.3%) |
| Onset-to-rehabilitation interval, days | 44.72 ± 15.34 | 44.59 ± 15.88 |
| Onset-to-aquatic-therapy interval, days | 66.44 ± 21.18 | 65.79 ± 21.83 |
| Baseline NIHSS | 7.70 ± 3.47 | 7.49 ± 3.60 |
| Baseline FMA-UE | 38.86 ± 12.56 | 39.11 ± 12.25 |
| Baseline FMA-LE | 19.53 ± 6.35 | 19.84 ± 6.40 |
| Baseline MBI | 47.60 ± 19.23 | 48.94 ± 19.02 |
| Baseline FAC | 2.47 ± 1.06 | 2.45 ± 1.05 |
| Baseline Berg Balance Scale | 30.53 ± 9.95 | 31.19 ± 10.23 |
| MMSE | 25.66 ± 2.91 | 25.77 ± 2.89 |
| HAMD | 7.98 ± 4.48 | 7.73 ± 4.36 |
| Planned aquatic session duration, min | 32.82 ± 4.35 | 33.15 ± 4.23 |
| Planned aquatic frequency, sessions/week | 3.23 ± 0.81 | 3.20 ± 0.82 |
| Planned aquatic course, weeks | 8.58 ± 1.67 | 8.54 ± 1.57 |
| Initial underwater-treadmill speed, m/s | 0.67 ± 0.19 | 0.67 ± 0.19 |
| Target Borg rating | 12.49 ± 1.05 | 12.51 ± 1.06 |

**Supplementary Table S2A. Predictor missingness before imputation**

| Predictor | Dev. miss., n | Dev. miss., % | Val. miss., n | Val. miss., % | Overall miss., n | Overall miss., % | Reason | Excluded | Handling |
| --- | --- | --- | --- | --- | --- | --- | --- | --- | --- |
| HbA1c, % | 30 | 3.8% | 29 | 5.4% | 59 | 4.5% | test not performed | No | KNN-5 within fold |
| LDL cholesterol, mmol/L | 36 | 4.6% | 31 | 5.8% | 67 | 5.1% | test not performed | No | KNN-5 within fold |
| Motor-evoked-potential result | 63 | 8.0% | 41 | 7.7% | 104 | 7.9% | examination not completed or record unavailable | No | Most frequent category within fold |
| MMSE | 34 | 4.3% | 30 | 5.6% | 64 | 4.8% | record unavailable | No | KNN-5 within fold |
| HAMD | 47 | 6.0% | 28 | 5.2% | 75 | 5.7% | record unavailable | No | KNN-5 within fold |

**Supplementary Table S2B. Predictor dictionary, coding, timing, and model membership**

| Source predictor | Type | Window | A cand. | B cand. | Dev. miss., n | Val. miss., n | Coding | Sel. A | Sel. B |
| --- | --- | --- | --- | --- | --- | --- | --- | --- | --- |
| Age, years | Num | Pre-session | Y | Y | 0 | 0 | N | N | N |
| Sex | Cat | Pre-session | Y | Y | 0 | 0 | C | N | N |
| BMI, kg/m² | Num | Pre-session | Y | Y | 0 | 0 | N | N | N |
| Education, years | Num | Pre-session | Y | Y | 0 | 0 | N | N | N |
| Hypertension | Cat | Pre-session | Y | Y | 0 | 0 | C | N | N |
| Diabetes mellitus | Cat | Pre-session | Y | Y | 0 | 0 | C | N | N |
| Atrial fibrillation | Cat | Pre-session | Y | Y | 0 | 0 | C | N | N |
| Smoking history | Cat | Pre-session | Y | Y | 0 | 0 | C | N | N |
| Alcohol-use history | Cat | Pre-session | Y | Y | 0 | 0 | C | N | N |
| Systolic blood pressure, mmHg | Num | Pre-session | Y | Y | 0 | 0 | N | N | N |
| Diastolic blood pressure, mmHg | Num | Pre-session | Y | Y | 0 | 0 | N | N | N |
| HbA1c, % | Num | Pre-session | Y | Y | 30 | 29 | N | N | N |
| LDL cholesterol, mmol/L | Num | Pre-session | Y | Y | 36 | 31 | N | N | N |
| C-reactive protein, mg/L | Num | Pre-session | Y | Y | 0 | 0 | N | N | N |
| Albumin, g/L | Num | Pre-session | Y | Y | 0 | 0 | N | N | N |
| Haemoglobin, g/L | Num | Pre-session | Y | Y | 0 | 0 | N | N | N |
| Stroke type | Cat | Pre-session | Y | Y | 0 | 0 | C | N | N |
| Lesion side | Cat | Pre-session | Y | Y | 0 | 0 | C | N | N |
| Lesion location | Cat | Pre-session | Y | Y | 0 | 0 | C | N | N |
| Corticospinal-tract involvement | Cat | Pre-session | Y | Y | 0 | 0 | C | N | N |
| Motor-evoked-potential result | Cat | Pre-session | Y | Y | 63 | 41 | C | N | N |
| Onset-to-rehabilitation interval, days | Num | Pre-session | Y | Y | 0 | 0 | N | Y | Y |
| Onset-to-aquatic-therapy interval, days | Num | Pre-session | Y | Y | 0 | 0 | N | N | N |
| Baseline NIHSS | Num | Pre-session | Y | Y | 0 | 0 | N | N | N |
| Baseline FMA-UE | Num | Pre-session | Y | Y | 0 | 0 | N | Y | Y |
| Baseline FMA-LE | Num | Pre-session | Y | Y | 0 | 0 | N | Y | Y |
| Baseline MBI | Num | Pre-session | Y | Y | 0 | 0 | N | N | N |
| Baseline FAC | Num | Pre-session | Y | Y | 0 | 0 | N | N | N |
| Baseline Berg Balance Scale | Num | Pre-session | Y | Y | 0 | 0 | N | N | N |
| Modified Ashworth Scale | Num | Pre-session | Y | Y | 0 | 0 | N | N | N |
| Pain VAS | Num | Pre-session | Y | Y | 0 | 0 | N | N | N |
| MMSE | Num | Pre-session | Y | Y | 34 | 30 | N | N | N |
| HAMD | Num | Pre-session | Y | Y | 47 | 28 | N | N | N |
| Shoulder pain or subluxation | Cat | Pre-session | Y | Y | 0 | 0 | C | N | N |
| Dysphagia | Cat | Pre-session | Y | Y | 0 | 0 | C | N | N |
| Dysarthria | Cat | Pre-session | Y | Y | 0 | 0 | C | N | N |
| Spasticity | Cat | Pre-session | Y | Y | 0 | 0 | C | N | N |
| Pain | Cat | Pre-session | Y | Y | 0 | 0 | C | N | N |
| Cognitive impairment | Cat | Pre-session | Y | Y | 0 | 0 | C | N | N |
| Depression or anxiety | Cat | Pre-session | Y | Y | 0 | 0 | C | N | N |
| Primary mobility method or aid | Cat | Pre-session | Y | Y | 0 | 0 | C | N | N |
| Antiplatelet therapy | Cat | Pre-session | Y | Y | 0 | 0 | C | N | N |
| Anticoagulant therapy | Cat | Pre-session | Y | Y | 0 | 0 | C | N | N |
| Statin therapy | Cat | Pre-session | Y | Y | 0 | 0 | C | N | N |
| Antihypertensive therapy | Cat | Pre-session | Y | Y | 0 | 0 | C | N | N |
| Glucose-lowering therapy | Cat | Pre-session | Y | Y | 0 | 0 | C | N | N |
| Planned aquatic session duration, min | Num | Pre-session | Y | Y | 0 | 0 | N | N | N |
| Planned aquatic frequency, sessions/week | Num | Pre-session | Y | Y | 0 | 0 | N | Y | Y |
| Planned aquatic course, weeks | Num | Pre-session | Y | Y | 0 | 0 | N | Y | Y |
| Initial underwater-treadmill speed, m/s | Num | Pre-session | Y | Y | 0 | 0 | N | N | N |
| Target Borg rating | Num | Pre-session | Y | Y | 0 | 0 | N | N | N |
| Prescribed water depth, cm | Num | Pre-session | Y | Y | 0 | 0 | N | N | N |
| Prescribed water temperature, °C | Num | Pre-session | Y | Y | 0 | 0 | N | N | N |
| Prescribed flow/vortex intensity | Cat | Pre-session | Y | Y | 0 | 0 | C | N | N |
| Planned PT frequency, sessions/week | Num | Pre-session | Y | Y | 0 | 0 | N | N | N |
| Planned PT session duration, min | Num | Pre-session | Y | Y | 0 | 0 | N | N | N |
| Planned OT frequency, sessions/week | Num | Pre-session | Y | Y | 0 | 0 | N | N | N |
| Planned OT session duration, min | Num | Pre-session | Y | Y | 0 | 0 | N | N | N |
| Planned ST frequency, sessions/week | Num | Pre-session | Y | Y | 0 | 0 | N | N | N |
| Planned ST session duration, min | Num | Pre-session | Y | Y | 0 | 0 | N | N | N |
| Planned gait-training frequency, sessions/week | Num | Pre-session | Y | Y | 0 | 0 | N | N | N |
| Planned gait-training session duration, min | Num | Pre-session | Y | Y | 0 | 0 | N | N | N |
| Planned balance-training frequency, sessions/week | Num | Pre-session | Y | Y | 0 | 0 | N | N | N |
| Planned balance-training session duration, min | Num | Pre-session | Y | Y | 0 | 0 | N | N | N |
| Planned robot/BWS gait frequency, sessions/week | Num | Pre-session | Y | Y | 0 | 0 | N | N | N |
| Planned robot/BWS gait session duration, min | Num | Pre-session | Y | Y | 0 | 0 | N | N | N |
| Planned NMES frequency, sessions/week | Num | Pre-session | Y | Y | 0 | 0 | N | N | N |
| Planned NMES session duration, min | Num | Pre-session | Y | Y | 0 | 0 | N | N | N |
| Planned acupuncture frequency, sessions/week | Num | Pre-session | Y | Y | 0 | 0 | N | N | N |
| Planned acupuncture session duration, min | Num | Pre-session | Y | Y | 0 | 0 | N | N | N |
| Planned mirror-therapy frequency, sessions/week | Num | Pre-session | Y | Y | 0 | 0 | N | N | N |
| Planned mirror-therapy session duration, min | Num | Pre-session | Y | Y | 0 | 0 | N | N | N |
| Planned conventional-exercise frequency, sessions/week | Num | Pre-session | Y | Y | 0 | 0 | N | N | N |
| Planned conventional-exercise session duration, min | Num | Pre-session | Y | Y | 0 | 0 | N | N | N |
| Planned home-training guidance frequency, sessions/week | Num | Pre-session | Y | Y | 0 | 0 | N | N | N |
| Planned home-training guidance duration, min | Num | Pre-session | Y | Y | 0 | 0 | N | N | N |
| Aquatic sessions completed by day 14 | Num | Day 14* | N | Y | 0 | 0 | N | N | Y |
| Mean aquatic session duration by day 14, min | Num | Day 14* | N | Y | 0 | 0 | N | N | N |
| Mean underwater-treadmill speed by day 14, m/s | Num | Day 14* | N | Y | 0 | 0 | N | N | Y |
| Mean Borg rating by day 14 | Num | Day 14* | N | Y | 0 | 0 | N | N | N |
| Mean water depth by day 14, cm | Num | Day 14* | N | Y | 0 | 0 | N | N | N |
| Mean water temperature by day 14, °C | Num | Day 14* | N | Y | 0 | 0 | N | N | N |
| Modal flow/vortex intensity by day 14 | Cat | Day 14* | N | Y | 0 | 0 | C | N | N |
| PT sessions completed by day 14 | Num | Day 14* | N | Y | 0 | 0 | N | N | N |
| Mean PT session duration by day 14, min | Num | Day 14* | N | Y | 0 | 0 | N | N | N |
| OT sessions completed by day 14 | Num | Day 14* | N | Y | 0 | 0 | N | N | Y |
| Mean OT session duration by day 14, min | Num | Day 14* | N | Y | 0 | 0 | N | N | N |
| Gait-training sessions completed by day 14 | Num | Day 14* | N | Y | 0 | 0 | N | N | N |
| Mean gait-training duration by day 14, min | Num | Day 14* | N | Y | 0 | 0 | N | N | Y |
| Balance-training sessions completed by day 14 | Num | Day 14* | N | Y | 0 | 0 | N | N | N |
| Mean balance-training duration by day 14, min | Num | Day 14* | N | Y | 0 | 0 | N | N | N |
| Robot/BWS gait sessions completed by day 14 | Num | Day 14* | N | Y | 0 | 0 | N | N | N |
| Mean robot/BWS gait duration by day 14, min | Num | Day 14* | N | Y | 0 | 0 | N | N | N |
| NMES sessions completed by day 14 | Num | Day 14* | N | Y | 0 | 0 | N | N | N |
| Mean NMES duration by day 14, min | Num | Day 14* | N | Y | 0 | 0 | N | N | Y |

Note: N = continuous variable, KNN-5 imputation within the training fold and standardisation; C = categorical variable, training-fold mode imputation and one-hot coding with all levels retained (no dropped reference category). Pre-session = available before the first aquatic session. Day 14* = dated on or before the day-14 landmark, with allowable reporting window days 12–16.

**Supplementary Table S3A. Feature-number cross-validation curves**

| Retained encoded features | Mean CV AUC | SD | SE | Model |
| --- | --- | --- | --- | --- |
| 5 | 0.8274 | 0.0310 | 0.0139 | Model A |
| 10 | 0.8311 | 0.0364 | 0.0163 | Model A |
| 15 | 0.8315 | 0.0419 | 0.0187 | Model A |
| 20 | 0.8219 | 0.0396 | 0.0177 | Model A |
| 25 | 0.8281 | 0.0410 | 0.0184 | Model A |
| 30 | 0.8210 | 0.0357 | 0.0160 | Model A |
| all | 0.8120 | 0.0499 | 0.0223 | Model A |
| 5 | 0.8231 | 0.0232 | 0.0104 | Model B |
| 10 | 0.8376 | 0.0214 | 0.0096 | Model B |
| 15 | 0.8352 | 0.0339 | 0.0152 | Model B |
| 20 | 0.8354 | 0.0333 | 0.0149 | Model B |
| 25 | 0.8285 | 0.0405 | 0.0181 | Model B |
| 30 | 0.8234 | 0.0411 | 0.0184 | Model B |
| all | 0.8020 | 0.0460 | 0.0206 | Model B |

Note: the unrounded maximum mean AUC occurred at 15 features for Model A and 10 features for Model B; the one-standard-error rule selected 5 and 10 features, respectively.

**Supplementary Table S3B. RFE selection stability across 30 stratified bootstrap samples**

| Model | Predictor | Selected, n/30 | Selection frequency |
| --- | --- | --- | --- |
| Model A | Baseline FMA-UE | 30 | 1.000 |
| Model A | Planned aquatic frequency, sessions/week | 30 | 1.000 |
| Model A | Planned aquatic course, weeks | 30 | 1.000 |
| Model A | Initial underwater-treadmill speed, m/s | 15 | 0.500 |
| Model A | Baseline FMA-LE | 13 | 0.433 |
| Model A | Baseline MBI | 8 | 0.267 |
| Model A | Onset-to-rehabilitation interval, days | 5 | 0.167 |
| Model A | Systolic blood pressure, mmHg | 3 | 0.100 |
| Model A | HbA1c, % | 3 | 0.100 |
| Model A | Haemoglobin, g/L | 2 | 0.067 |
| Model A | Planned acupuncture session duration, min | 2 | 0.067 |
| Model A | C-reactive protein, mg/L | 1 | 0.033 |
| Model A | HAMD | 1 | 0.033 |
| Model A | LDL cholesterol, mmol/L | 1 | 0.033 |
| Model A | Planned OT frequency, sessions/week | 1 | 0.033 |
| Model A | Education, years | 1 | 0.033 |
| Model A | Planned gait-training session duration, min | 1 | 0.033 |
| Model A | Pain VAS | 1 | 0.033 |
| Model A | Albumin, g/L | 1 | 0.033 |
| Model A | Planned NMES session duration, min | 1 | 0.033 |
| Model B | Aquatic sessions completed by day 14 | 30 | 1.000 |
| Model B | Baseline FMA-UE | 30 | 1.000 |
| Model B | Planned aquatic course, weeks | 30 | 1.000 |
| Model B | Planned aquatic frequency, sessions/week | 28 | 0.933 |
| Model B | Mean gait-training duration by day 14, min | 21 | 0.700 |
| Model B | Mean underwater-treadmill speed by day 14, m/s | 20 | 0.667 |
| Model B | Mean NMES duration by day 14, min | 16 | 0.533 |
| Model B | Baseline FMA-LE | 13 | 0.433 |
| Model B | Initial underwater-treadmill speed, m/s | 12 | 0.400 |
| Model B | OT sessions completed by day 14 | 11 | 0.367 |
| Model B | Onset-to-rehabilitation interval, days | 11 | 0.367 |
| Model B | Mean PT session duration by day 14, min | 9 | 0.300 |
| Model B | Baseline MBI | 9 | 0.300 |
| Model B | HbA1c, % | 9 | 0.300 |
| Model B | Planned acupuncture session duration, min | 6 | 0.200 |
| Model B | Onset-to-aquatic-therapy interval, days | 5 | 0.167 |
| Model B | Planned OT frequency, sessions/week | 3 | 0.100 |
| Model B | Mean aquatic session duration by day 14, min | 3 | 0.100 |
| Model B | Mean balance-training duration by day 14, min | 3 | 0.100 |
| Model B | Education, years | 3 | 0.100 |
| Model B | Albumin, g/L | 3 | 0.100 |
| Model B | BMI, kg/m² | 2 | 0.067 |
| Model B | HAMD | 2 | 0.067 |
| Model B | Planned ST session duration, min | 2 | 0.067 |
| Model B | Balance-training sessions completed by day 14 | 2 | 0.067 |
| Model B | Mean OT session duration by day 14, min | 2 | 0.067 |
| Model B | Systolic blood pressure, mmHg | 2 | 0.067 |
| Model B | LDL cholesterol, mmol/L | 1 | 0.033 |
| Model B | Mean water depth by day 14, cm | 1 | 0.033 |
| Model B | Mean water temperature by day 14, °C | 1 | 0.033 |
| Model B | Robot/BWS gait sessions completed by day 14 | 1 | 0.033 |
| Model B | Mean robot/BWS gait duration by day 14, min | 1 | 0.033 |
| Model B | Baseline Berg Balance Scale | 1 | 0.033 |
| Model B | Baseline NIHSS | 1 | 0.033 |
| Model B | Planned home-training guidance duration, min | 1 | 0.033 |
| Model B | Age, years | 1 | 0.033 |
| Model B | Planned robot/BWS gait session duration, min | 1 | 0.033 |
| Model B | Planned gait-training session duration, min | 1 | 0.033 |
| Model B | Pain VAS | 1 | 0.033 |
| Model B | Haemoglobin, g/L | 1 | 0.033 |

**Supplementary Table S4A. Performance by baseline FMA severity and ceiling-sensitive subset**

| Model | Baseline-FMA subgroup | n | Events | AUC | AUC CI low | AUC CI high | Sensitivity | Specificity | PPV | NPV | F1 | Brier score |
| --- | --- | --- | --- | --- | --- | --- | --- | --- | --- | --- | --- | --- |
| Model A | Severe/very severe (FMA 0–55) | 222 | 80 | 0.800 | 0.741 | 0.860 | 0.875 | 0.507 | 0.500 | 0.878 | 0.636 | 0.186 |
| Model A | Moderate (FMA 56–79) | 257 | 63 | 0.816 | 0.757 | 0.870 | 0.794 | 0.716 | 0.476 | 0.914 | 0.595 | 0.160 |
| Model A | Mild (FMA 80–100) | 55 | 25 | 0.825 | 0.708 | 0.923 | 0.960 | 0.633 | 0.686 | 0.950 | 0.800 | 0.172 |
| Model A | Baseline FMA >90 | 17 | 17 | — | — | — | 1.000 | — | 1.000 | — | 1.000 | 0.110 |
| Model A | Ceiling-sensitive cohort (FMA ≤90) | 517 | 151 | 0.807 | 0.767 | 0.848 | 0.841 | 0.628 | 0.483 | 0.906 | 0.614 | 0.174 |
| Model B | Severe/very severe (FMA 0–55) | 222 | 80 | 0.797 | 0.738 | 0.856 | 0.825 | 0.592 | 0.532 | 0.857 | 0.647 | 0.187 |
| Model B | Moderate (FMA 56–79) | 257 | 63 | 0.823 | 0.761 | 0.882 | 0.698 | 0.784 | 0.512 | 0.889 | 0.591 | 0.159 |
| Model B | Mild (FMA 80–100) | 55 | 25 | 0.797 | 0.675 | 0.903 | 0.840 | 0.567 | 0.618 | 0.810 | 0.712 | 0.190 |
| Model B | Baseline FMA >90 | 17 | 17 | — | — | — | 0.882 | — | 1.000 | 0.000 | 0.938 | 0.152 |
| Model B | Ceiling-sensitive cohort (FMA ≤90) | 517 | 151 | 0.805 | 0.763 | 0.842 | 0.768 | 0.691 | 0.507 | 0.878 | 0.611 | 0.175 |

**Supplementary Table S4B. Subgroup calibration point estimates**

| Model | Baseline-FMA subgroup | Calibration slope | Joint intercept | CITL | MCE |
| --- | --- | --- | --- | --- | --- |
| Model A | Severe/very severe (FMA 0–55) | 1.412 | -0.614 | -0.560 | 0.260 |
| Model A | Moderate (FMA 56–79) | 1.336 | -0.618 | -0.694 | 0.275 |
| Model A | Mild (FMA 80–100) | 1.355 | -0.460 | -0.346 | 0.408 |
| Model A | Baseline FMA >90 | — | — | — | — |
| Model A | Ceiling-sensitive cohort (FMA ≤90) | 1.311 | -0.671 | -0.676 | 0.267 |
| Model B | Severe/very severe (FMA 0–55) | 1.542 | -0.527 | -0.502 | 0.240 |
| Model B | Moderate (FMA 56–79) | 1.778 | -0.467 | -0.677 | 0.242 |
| Model B | Mild (FMA 80–100) | 1.474 | -0.254 | -0.198 | 0.311 |
| Model B | Baseline FMA >90 | — | — | — | — |
| Model B | Ceiling-sensitive cohort (FMA ≤90) | 1.567 | -0.580 | -0.633 | 0.239 |

**Supplementary Table S5A. Frozen threshold-specific validation performance**

| Model | Algorithm | Operating rule | Threshold | AUC | Accuracy | Sensitivity | Specificity | PPV | NPV | F1 | Brier score |
| --- | --- | --- | --- | --- | --- | --- | --- | --- | --- | --- | --- |
| Model A | Random Forest | Youden ref. | 0.435 | 0.818 | 0.713 | 0.839 | 0.656 | 0.528 | 0.899 | 0.648 | 0.172 |
| Model A | Random Forest | Sens. target ≥0.80 | 0.408 | 0.818 | 0.700 | 0.857 | 0.628 | 0.514 | 0.906 | 0.643 | 0.172 |
| Model A | Random Forest | Sens. target ≥0.85 | 0.380 | 0.818 | 0.669 | 0.893 | 0.566 | 0.485 | 0.920 | 0.629 | 0.172 |
| Model A | Random Forest | Sens. target ≥0.90 | 0.327 | 0.818 | 0.624 | 0.923 | 0.486 | 0.452 | 0.932 | 0.607 | 0.172 |
| Model B | Random Forest | Youden ref. | 0.438 | 0.814 | 0.719 | 0.786 | 0.689 | 0.537 | 0.875 | 0.638 | 0.174 |
| Model B | Random Forest | Sens. target ≥0.80 | 0.441 | 0.814 | 0.719 | 0.780 | 0.691 | 0.537 | 0.872 | 0.636 | 0.174 |
| Model B | Random Forest | Sens. target ≥0.85 | 0.404 | 0.814 | 0.710 | 0.869 | 0.637 | 0.523 | 0.914 | 0.653 | 0.174 |
| Model B | Random Forest | Sens. target ≥0.90 | 0.330 | 0.814 | 0.625 | 0.923 | 0.489 | 0.453 | 0.932 | 0.608 | 0.174 |

**Supplementary Table S5B. Confusion counts and calibration point estimates**

| Model | Operating rule | TP | FN | FP | TN | Calibration slope | Joint intercept | CITL | MCE |
| --- | --- | --- | --- | --- | --- | --- | --- | --- | --- |
| Model A | Youden ref. | 141 | 27 | 126 | 240 | 1.380 | -0.594 | -0.595 | 0.226 |
| Model A | Sens. target ≥0.80 | 144 | 24 | 136 | 230 | 1.380 | -0.594 | -0.595 | 0.226 |
| Model A | Sens. target ≥0.85 | 150 | 18 | 159 | 207 | 1.380 | -0.594 | -0.595 | 0.226 |
| Model A | Sens. target ≥0.90 | 155 | 13 | 188 | 178 | 1.380 | -0.594 | -0.595 | 0.226 |
| Model B | Youden ref. | 132 | 36 | 114 | 252 | 1.637 | -0.490 | -0.544 | 0.197 |
| Model B | Sens. target ≥0.80 | 131 | 37 | 113 | 253 | 1.637 | -0.490 | -0.544 | 0.197 |
| Model B | Sens. target ≥0.85 | 146 | 22 | 133 | 233 | 1.637 | -0.490 | -0.544 | 0.197 |
| Model B | Sens. target ≥0.90 | 155 | 13 | 187 | 179 | 1.637 | -0.490 | -0.544 | 0.197 |

**Supplementary Table S5C. Bootstrap confidence intervals**

| Model | Operating rule | AUC 95% CI | Accuracy 95% CI | Sensitivity 95% CI | Specificity 95% CI | PPV 95% CI | NPV 95% CI | F1 95% CI | Brier 95% CI |
| --- | --- | --- | --- | --- | --- | --- | --- | --- | --- |
| Model A | Youden ref. | 0.777–0.853 | 0.674–0.751 | 0.780–0.893 | 0.609–0.700 | 0.490–0.567 | 0.864–0.930 | 0.607–0.688 | 0.160–0.186 |
| Model A | Sens. target ≥0.80 | 0.777–0.853 | 0.661–0.736 | 0.804–0.905 | 0.582–0.672 | 0.479–0.551 | 0.870–0.938 | 0.603–0.680 | 0.160–0.186 |
| Model A | Sens. target ≥0.85 | 0.777–0.853 | 0.633–0.704 | 0.845–0.935 | 0.516–0.612 | 0.457–0.517 | 0.886–0.953 | 0.596–0.661 | 0.160–0.186 |
| Model A | Sens. target ≥0.90 | 0.777–0.853 | 0.586–0.659 | 0.881–0.958 | 0.434–0.533 | 0.426–0.479 | 0.894–0.964 | 0.578–0.635 | 0.160–0.186 |
| Model B | Youden ref. | 0.774–0.850 | 0.682–0.757 | 0.720–0.845 | 0.642–0.735 | 0.496–0.581 | 0.841–0.905 | 0.591–0.680 | 0.163–0.186 |
| Model B | Sens. target ≥0.80 | 0.774–0.850 | 0.682–0.755 | 0.714–0.839 | 0.645–0.735 | 0.496–0.581 | 0.838–0.902 | 0.590–0.680 | 0.163–0.186 |
| Model B | Sens. target ≥0.85 | 0.774–0.850 | 0.674–0.745 | 0.815–0.917 | 0.590–0.680 | 0.489–0.559 | 0.880–0.943 | 0.615–0.691 | 0.163–0.186 |
| Model B | Sens. target ≥0.90 | 0.774–0.850 | 0.588–0.659 | 0.881–0.958 | 0.440–0.536 | 0.428–0.478 | 0.897–0.964 | 0.579–0.636 | 0.163–0.186 |

**Supplementary Table S5D. Model-level calibration estimates with 95% bootstrap confidence intervals**

| Model | Brier score | Calibration slope | Joint intercept | Calibration-in-the-large | MCE |
| --- | --- | --- | --- | --- | --- |
| Model A | 0.172 (0.160–0.186) | 1.380 (1.118–1.663) | −0.594 (−0.708 to −0.485) | −0.595 (−0.674 to −0.515) | 0.226 (0.168–0.317) |
| Model B | 0.174 (0.163–0.186) | 1.637 (1.343–1.976) | −0.490 (−0.596 to −0.377) | −0.544 (−0.607 to −0.480) | 0.197 (0.191–0.313) |

**Supplementary Table S6A. Candidate-algorithm development cross-validation**

| Algorithm | Development CV AUC | Selected hyperparameters | Model | Selected feature count |
| --- | --- | --- | --- | --- |
| Random Forest | 0.8290 | max_depth=5; min_samples_split=5; n_estimators=500 | Model A | 5 |
| ExtraTrees | 0.8272 | max_depth=5; min_samples_split=2; n_estimators=200 | Model A | 5 |
| XGBoost | 0.8269 | learning_rate=0.01; max_depth=3; n_estimators=500 | Model A | 5 |
| Logistic regression | 0.8069 | C=0.1; penalty=l2 | Model A | 5 |
| KNN | 0.8055 | n_neighbors=15; weights=distance | Model A | 5 |
| Random Forest | 0.8384 | max_depth=5; min_samples_split=5; n_estimators=500 | Model B | 10 |
| ExtraTrees | 0.8346 | max_depth=10; min_samples_split=5; n_estimators=500 | Model B | 10 |
| XGBoost | 0.8338 | learning_rate=0.01; max_depth=3; n_estimators=500 | Model B | 10 |
| Logistic regression | 0.8157 | C=1.0; penalty=l1 | Model B | 10 |
| KNN | 0.7977 | n_neighbors=15; weights=distance | Model B | 10 |

**Supplementary Table S6B. Temporal-validation discrimination and classification performance of all candidate algorithms**

| Model | Algorithm | Frozen cut-off | AUC (95% CI) | Accuracy (95% CI) | Sensitivity (95% CI) | Specificity (95% CI) | PPV (95% CI) | NPV (95% CI) | F1 (95% CI) | Brier (95% CI) |
| --- | --- | --- | --- | --- | --- | --- | --- | --- | --- | --- |
| Model A | Random Forest | 0.435 | 0.818 (0.777–0.853) | 0.713 (0.674–0.751) | 0.839 (0.780–0.893) | 0.656 (0.609–0.700) | 0.528 (0.490–0.567) | 0.899 (0.864–0.930) | 0.648 (0.607–0.688) | 0.172 (0.160–0.186) |
| Model A | ExtraTrees | 0.481 | 0.804 (0.761–0.842) | 0.706 (0.667–0.743) | 0.792 (0.726–0.845) | 0.667 (0.620–0.708) | 0.522 (0.480–0.561) | 0.875 (0.839–0.907) | 0.629 (0.582–0.670) | 0.186 (0.175–0.199) |
| Model A | XGBoost | 0.457 | 0.811 (0.772–0.847) | 0.730 (0.693–0.766) | 0.815 (0.756–0.863) | 0.691 (0.645–0.735) | 0.548 (0.508–0.590) | 0.891 (0.858–0.919) | 0.656 (0.612–0.696) | 0.174 (0.159–0.190) |
| Model A | Logistic regression | 0.569 | 0.775 (0.731–0.814) | 0.706 (0.668–0.742) | 0.613 (0.542–0.685) | 0.749 (0.705–0.790) | 0.528 (0.476–0.580) | 0.808 (0.778–0.837) | 0.567 (0.511–0.617) | 0.195 (0.180–0.213) |
| Model A | KNN | 0.298 | 0.769 (0.726–0.812) | 0.706 (0.667–0.742) | 0.714 (0.643–0.780) | 0.702 (0.656–0.743) | 0.524 (0.479–0.567) | 0.843 (0.810–0.875) | 0.605 (0.554–0.650) | 0.177 (0.161–0.194) |
| Model B | Random Forest | 0.438 | 0.814 (0.774–0.850) | 0.719 (0.682–0.757) | 0.786 (0.720–0.845) | 0.689 (0.642–0.735) | 0.537 (0.496–0.581) | 0.875 (0.841–0.905) | 0.638 (0.591–0.680) | 0.174 (0.163–0.186) |
| Model B | ExtraTrees | 0.479 | 0.795 (0.751–0.833) | 0.723 (0.685–0.762) | 0.667 (0.595–0.732) | 0.749 (0.705–0.792) | 0.549 (0.500–0.605) | 0.830 (0.798–0.861) | 0.602 (0.546–0.654) | 0.175 (0.162–0.190) |
| Model B | XGBoost | 0.463 | 0.806 (0.767–0.841) | 0.723 (0.685–0.760) | 0.756 (0.685–0.815) | 0.708 (0.664–0.751) | 0.543 (0.500–0.588) | 0.863 (0.830–0.893) | 0.632 (0.586–0.675) | 0.174 (0.159–0.190) |
| Model B | Logistic regression | 0.446 | 0.781 (0.738–0.818) | 0.685 (0.644–0.725) | 0.774 (0.708–0.833) | 0.645 (0.596–0.694) | 0.500 (0.460–0.543) | 0.861 (0.824–0.894) | 0.607 (0.562–0.648) | 0.189 (0.173–0.208) |
| Model B | KNN | 0.331 | 0.756 (0.711–0.795) | 0.684 (0.644–0.721) | 0.577 (0.506–0.649) | 0.732 (0.683–0.773) | 0.497 (0.446–0.550) | 0.791 (0.760–0.821) | 0.534 (0.477–0.590) | 0.176 (0.163–0.190) |

**Supplementary Table S6C. Temporal-validation calibration of all candidate algorithms**

| Model | Algorithm | Brier (95% CI) | Calibration slope (95% CI) | Joint intercept (95% CI) | CITL (95% CI) | MCE (95% CI) |
| --- | --- | --- | --- | --- | --- | --- |
| Model A | Random Forest | 0.172 (0.160–0.186) | 1.380 (1.118–1.663) | −0.594 (−0.708 to −0.485) | −0.595 (−0.674 to −0.515) | 0.226 (0.168–0.317) |
| Model A | ExtraTrees | 0.186 (0.175–0.199) | 1.649 (1.319–2.045) | −0.711 (−0.820 to −0.609) | −0.682 (−0.742 to −0.618) | 0.265 (0.222–0.352) |
| Model A | XGBoost | 0.174 (0.159–0.190) | 1.042 (0.839–1.265) | −0.676 (−0.795 to −0.572) | −0.671 (−0.776 to −0.569) | 0.257 (0.184–0.326) |
| Model A | Logistic regression | 0.195 (0.180–0.213) | 0.995 (0.795–1.215) | −0.788 (−0.902 to −0.690) | −0.789 (−0.882 to −0.699) | 0.291 (0.234–0.400) |
| Model A | KNN | 0.177 (0.161–0.194) | 0.370 (0.185–0.689) | −0.269 (−0.431 to −0.034) | 0.167 (0.042 to 0.282) | 0.119 (0.093–0.273) |
| Model B | Random Forest | 0.174 (0.163–0.186) | 1.637 (1.343–1.976) | −0.490 (−0.596 to −0.377) | −0.544 (−0.607 to −0.480) | 0.197 (0.191–0.313) |
| Model B | ExtraTrees | 0.175 (0.162–0.190) | 1.117 (0.867–1.403) | −0.480 (−0.575 to −0.383) | −0.490 (−0.575 to −0.410) | 0.201 (0.165–0.315) |
| Model B | XGBoost | 0.174 (0.159–0.190) | 1.036 (0.852–1.254) | −0.593 (−0.695 to −0.491) | −0.593 (−0.689 to −0.496) | 0.185 (0.162–0.370) |
| Model B | Logistic regression | 0.189 (0.173–0.208) | 0.892 (0.723–1.097) | −0.716 (−0.828 to −0.624) | −0.736 (−0.843 to −0.630) | 0.223 (0.215–0.402) |
| Model B | KNN | 0.176 (0.163–0.190) | 0.727 (0.387–1.066) | −0.013 (−0.273 to 0.227) | 0.185 (0.086 to 0.272) | 0.127 (0.092–0.254) |

**Supplementary Table S7. Sensitivity analysis using ΔFMA-LE <6 points as the alternative outcome**

| Model | Algorithm | AUC | Accuracy | Sensitivity | Specificity | Brier score |
| --- | --- | --- | --- | --- | --- | --- |
| Model A | Random Forest | 0.781 | 0.715 | 0.682 | 0.743 | 0.190 |
| Model B | Random Forest | 0.779 | 0.700 | 0.595 | 0.788 | 0.191 |

Supplementary Figure S1. Numeric correlation
